# Engraftment of essential functions through multiple fecal microbiota transplants in chronic antibiotic-resistant pouchitis—a case study using metatranscriptomics

**DOI:** 10.1186/s40168-023-01713-9

**Published:** 2023-12-01

**Authors:** Zhi-Luo Deng, Dietmar H. Pieper, Andreas Stallmach, Arndt Steube, Marius Vital, Michael Reck, Irene Wagner-Döbler

**Affiliations:** 1 Group Computational Biology for Infection Research, Helmholtz Center for Infection Research, Brunswick, Germany; 2 Group Microbial Interactions and Processes, Helmholtz Center for Infection Research, Brunswick, Germany; 3 Department of Internal Medicine IV (Gastroenterology, Hepatology, and Infectious Diseases), Jena University Hospital, Jena, Germany; 4 Institute for Medical Microbiology and Hospital Epidemiology, Hannover Medical School, Hannover, Germany; 5 Group Microbial Communication, Helmholtz Center for Infection Research, Brunswick, Germany; 6 TÜV Rheinland, Cologne, Germany; 7 Institute of Microbiology, Technical University of Braunschweig, Brunswick, Germany

**Keywords:** Metatranscriptome, Fecal microbiota transplantation, Pouchitis, Engraftment, Activity alterations, Butyrate biosynthesis, Bile acid metabolism, *Faecalibacterium prausnitzii*

## Abstract

**Background:**

Ileal pouch-anal anastomosis (IPAA) is the standard of care after total proctocolectomy for ulcerative colitis (UC). Around 50% of patients will experience pouchitis, an idiopathic inflammatory condition. Antibiotics are the backbone of treatment of pouchitis; however, antibiotic-resistant pouchitis develops in 5–10% of those patients. It has been shown that fecal microbiota transplantation (FMT) is an effective treatment for UC, but results for FMT antibiotic-resistant pouchitis are inconsistent.

**Methods:**

To uncover which metabolic activities were transferred to the recipients during FMT and helped the remission, we performed a longitudinal case study of the gut metatranscriptomes from three patients and their donors. The patients were treated by two to three FMTs, and stool samples were analyzed for up to 140 days.

**Results:**

Reduced expression in pouchitis patients compared to healthy donors was observed for genes involved in biosynthesis of amino acids, cofactors, and B vitamins. An independent metatranscriptome dataset of UC patients showed a similar result. Other functions including biosynthesis of butyrate, metabolism of bile acids, and tryptophan were also much lower expressed in pouchitis. After FMT, these activities transiently increased, and the overall metatranscriptome profiles closely mirrored those of the respective donors with notable fluctuations during the subsequent weeks. The levels of the clinical marker fecal calprotectin were concordant with the metatranscriptome data. *Faecalibacterium prausnitzii* represented the most active species contributing to butyrate synthesis via the acetyl-CoA pathway. Remission occurred after the last FMT in all patients and was characterized by a microbiota activity profile distinct from donors in two of the patients.

**Conclusions:**

Our study demonstrates the clear but short-lived activity engraftment of donor microbiota, particularly the butyrate biosynthesis after each FMT. The data suggest that FMT triggers shifts in the activity of patient microbiota towards health which need to be repeated to reach critical thresholds. As a case study, these insights warrant cautious interpretation, and validation in larger cohorts is necessary for generalized applications. In the long run, probiotics with high taxonomic diversity consisting of well characterized strains could replace FMT to avoid the costly screening of donors and the risk of transferring unwanted genetic material.

Video Abstract

**Supplementary Information:**

The online version contains supplementary material available at 10.1186/s40168-023-01713-9.

## Background

Fecal microbiota transplantation (FMT) is a novel therapy, whereby the fecal microbiota of healthy donors are transferred to seriously ill recipients [1]. It is highly successful for recurrent infections by *Clostridioides difficile* (CDI) [2, 3]. Many other indications for FMT have been tested, and a meta-analysis of the current literature reported that FMT can also be successful for ulcerative colitis (UC) [4].

UC, one of two major types of inflammatory bowel disease (IBD), is a global disease with a rising worldwide prevalence [5]. UC is a lifelong illness that has a profound impact on patients. The current concept on UC etiology highlights a complex interplay of factors encompassing dysregulation of intestinal microbiota and immunity, genetic makeup, environmental and lifestyle factors, and drive chronic intestinal inflammation [6]. In the past few decades, substantial progress has revolutionized medical treatment approaches for UC. However, a significant proportion of patients with UC require surgical intervention. Restorative proctocolectomy with ileal pouch-anal anastomosis (IPAA) is the surgical treatment of choice for UC refractory to medical treatment or with malignant transformations. In patients with IPAA, pouchitis, a mucosal inflammation of the ileal reservoir, is the most common long-term complication with a 10-year cumulative incidence between 24 and 59% [7]. The symptoms of acute pouchitis are characterized by an increased frequency of stool, malaise, abdominal cramps, rectal bleeding, and sometimes fever.

Antibiotic treatment (ABT) in pouchitis is initially successful, but often inflammation becomes resistant to ABT, leaving FMT as an important final option. Reports about the outcome of FMT in chronic, antibiotic-resistant recurrent pouchitis patients are rare. Currently, there are mostly case reports and studies with very few patients available, and the results are variable [8–15]. A prospective, multicenter, double-blind, randomized, controlled trial on the efficiency of FMT for chronic recurrent pouchitis is underway [16, 17].

Stool microbiota of patients with IBD are the most extensively studied dysbiotic microbial communities to date. As part of the Human Microbiome Project, multi-omics datasets from several clinical cohorts were analyzed for the mechanisms and early markers of dysbiosis with the aim of developing a systems-level understanding of these diseases [18–20]. This hugely complex work, of which only a few findings are summarized below, provides an important baseline for the case studies reported here. Key findings in IBD, which were confirmed in these and several other studies, are loss of taxonomic and functional diversity, an increase in facultative anaerobes (e.g., *Escherichia coli*), and a decrease in obligately anaerobic producers of short-chain fatty acids (SCFA), in particular the butyrate producers *Faecalibacterium prausnitzii* and *Roseburia hominis*.

Although such huge cohort studies are invaluable bases for generalized conclusions, they cannot account for the enormous individual differences in microbiota composition and activity. Moreover, most studies rely on 16S rRNA gene sequencing, which provides a taxonomic fingerprint of the microbial community but little functional information or metagenome analyses which show the genetic potential but not the actual activity of the microbiota.

In our previous study [14], FMT was applied to five patients that suffered from chronic antibiotic refractory pouchitis. Remission occurred after two or three FMTs in four of the five patients. Analysis of the 16S rRNA gene amplicons had previously shown differences in donor microbiota engraftment between the subsequent FMTs [14]. Here, we analyzed the metatranscriptomes of the donors and three patients that experienced clinical remission and had a complete record of clinical data during FMT. These three case studies are to the best of our knowledge the first to use metatranscriptome analysis to follow the shifts in the gut microbiota activity before and after FMT and provide insights into which activity alterations contributed to remission.

We first analyzed the taxonomic and functional composition of the active communities in each patient and followed it during up to three FMTs and 140 days. Then we determined the functions that were provided to the patients during FMT by comparing health and pouchitis and identified the microbes driving those shifts.

Next, we focused on pathways for the gut metabolites butyrate, secondary bile acids, vitamin B12 (cobalamin), vitamin B6 (pyridoxine), and tryptophan-derived metabolites that have been shown to play crucial roles in the health of the gut and to alleviate its inflammation [21]. Based on their documented importance, we focused on pathways for these metabolites in health and disease and analyzed the differentially expressed genes and the contributions of bacterial taxa to these functions. Subsequently, we followed their expression during two to three FMTs in the patients.

These analyses showed that *F. prausnitzii* played an important role. It is the most active contributor to the biosynthesis of butyrate. Therefore, we followed the activity of *F. prausnitzii* in health and disease as well as during the whole treatment process in detail.

Our analysis shows that engraftment of donor microbiota in recipients clearly occurred but was not stable. The downregulated genes in pouchitis were restored in patients temporarily after FMT. Final remission required several FMTs and was characterized by an active community distinct from the donors and the patients before treatment in two of the three patients, while in the third one, the patient acquired a microbial community similar to that of the donor.

## Results

### Study design and clinical data

Three patients suffering from chronic antibiotic-resistant pouchitis were followed through two to three FMTs by obtaining stool samples. The three patients selected for this study were from our previously published clinical research [14], and their clinical data are shown in Table S1 sheet 1. We specifically chose these individuals for our research because they were the only ones with comprehensive records of both fecal calprotectin (FC) levels and pouchitis disease activity index (PDAI) scores. From all patients, two samples were collected prior to treatment, representing the diseased state. All patients received ABT before the first FMT. For metatranscriptomes, the patients were followed for 140 days (CG), 99 days (DW), and 55 days (JM) after the first FMT (Table S1 sheets 1–2). The FC levels were followed until 4–6 months after the last FMT (Table S1 sheets 1–2). Details on ABT and FMT procedure can be found in [14]. Before FMT, all patients presented with significantly elevated FC levels above 400 μg/g (Table S1 sheet 1). These levels noticeably dropped after FMT. Antibiotic-free remission for 3 to 4 months was observed for all three patients after the last FMT as reflected by their PDAI scores.

### Sequencing data and metatranscriptome analysis

We determined the metatranscriptomes of 49 stool samples. Seven samples from two different donors and 42 samples from three different patients were analyzed. The complete sample list can be found in Table S1 sheet 2. Sequencing generated 4,266,728,608 (~ 4.3 billion) reads in total. Of these, 50% represented ribosomal RNA. Human reads comprised 1.89%. Thus, the amount of putative mRNA reads available for analysis was ~ 2 billion reads, on average 41,849,255 ± 23,966,569 (41.8 million) reads per sample. Of these, 43.8% could be assigned a taxonomy by Kraken [22] using the Kraken standard database including NCBI RefSeq genomes of archaea, bacteria, fungi, human, plasmids, and viruses. To comprehensively analyze the activity and taxonomy of the metatranscriptome, we used the integrated catalogue of reference genes in the human gut microbiome database (IGC) which comprises 9,879,896 reference genes [23] for mapping. Using this database, 86.71% of all cleaned metatranscriptomic reads could be mapped with BWA-MEM [24]. This mapping rate was improved further to 87.91% by including additional nonredundant genes into the IGC database (see “Methods” for details, Figure S1A, and Table S1 sheet 3). To account for the expansion of the NCBI RefSeq database in recent years and because the taxonomy information of IGC does not include species level, its taxonomy assignment was updated using centrifuge [25] (see “Methods” for details). With this improved database, 70.35% reads were assigned to phylum level and 65.45% to genus. The downstream functional enrichment and activity engraftment analyses are illustrated in Figure S1B.

### Taxonomic composition and gene expression of the microbial communities

**Fig. 1:**
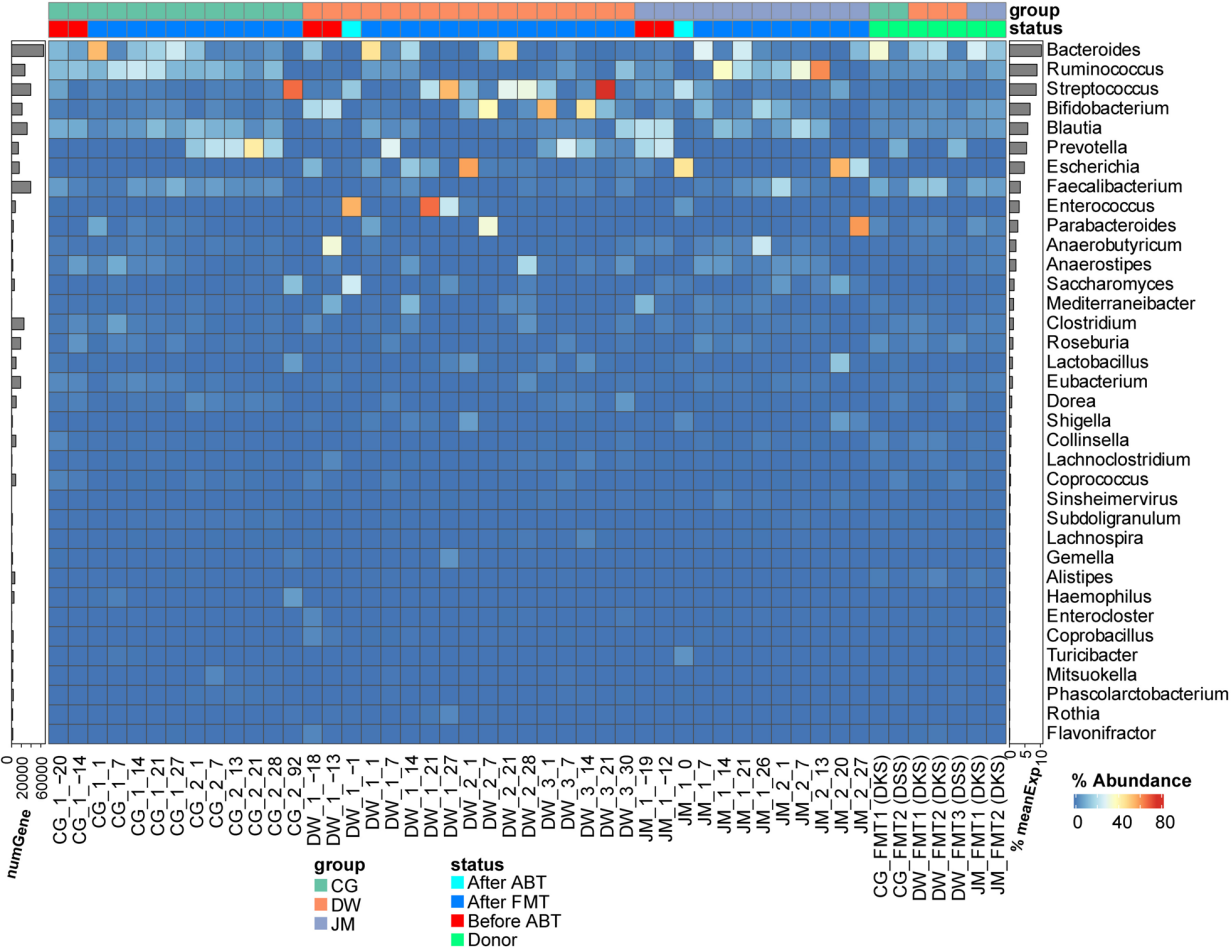
Heatmap of taxonomic composition of the gut community during FMT based on expressed genes on the genus level. Metatranscriptome reads were mapped to an improved IGC (Integrated Gene Catalogue) database to assign functional and taxonomic information (see “Methods”) and then grouped according to genera. Abundance was normalized by the total number of mapped reads per sample. Only genera with an average relative abundance > 0.1% across all samples are shown, which represented > 98% of all taxonomically assigned reads. Right side bar, mean of relative expression of all genes for this genus across all samples; left side bar, number of expressed genes from this genus across all samples. The two column annotation bars depict the patient groups (CG, DW, and JM) and the disease status. The status “before/after ABT” indicates before or after antibiotic treatment. More details can be found in “Methods”

The mapped reads were contributed by five main phyla, Actinobacteria, Ascomycota, Bacteroidetes, Firmicutes, and Proteobacteria (Table S1 sheet 4) of which Ascomycota is a fungal phylum. Figure 1 (heatmap and right bar) shows the taxonomic composition of transcripts on the level of genus across all sampling timepoints based on all reads mapped to genes with genus assignment. *Bacteroides* was most active, followed by *Ruminococcus*, *Streptococcus*, *Bifidobacterium*, *Blautia*, *Prevotella*, *Escherichia*, and *Faecalibacterium* (Fig. 1, Figure S2, Table S1 sheet 5). *Bacteroides* was also the most abundant genus in our previous study based on 16S rRNA gene amplicon sequencing (Figure S3), followed by *Prevotella*, *Streptococcus*, *Faecalibacterium*, and *Escherichia/Shigella*. According to the 16S rRNA gene amplicon sequencing data, S*treptococcus* and *Escherichia/Shigella* were abundant in patients before FMT but not present in any of the donors. *Faecalibacterium* was abundant in both donors but very low abundant in patients DW and JM before FMT.

In total, 815,055 unique genes were expressed with an average read count > 1 among all samples. They account for over 99% of mapped reads. Of the unique expressed genes, 65,991 were contributed by *Bacteroides*, 39,132 by *Faecalibacterium*, 39,035 by *Streptococcus*, 31,857 by *Blautia*, and then followed by *Ruminococcus*, *Clostridium* (sensu stricto), and *Bifidobacterium* (Fig. 1, left barplot). The cumulative rank abundance curve for all reads clustered according to genus showed that saturation was reached after 20 genera or less (Figure S4). Donors reached saturation latest and harbored the most diverse communities: an 80% coverage of all reads mapped to taxonomically assigned genes was reached in donor communities by 9.14 ± 1.07 genera, while in patients before treatment, such coverage was achieved by 6.00 ± 1.10. In patients after ABT and FMT, this coverage was achieved by 4.88 ± 2.06 genera. The result suggests that donor’s metatranscriptomes had higher taxonomy diversity (Figure S4).

**Fig. 2:**
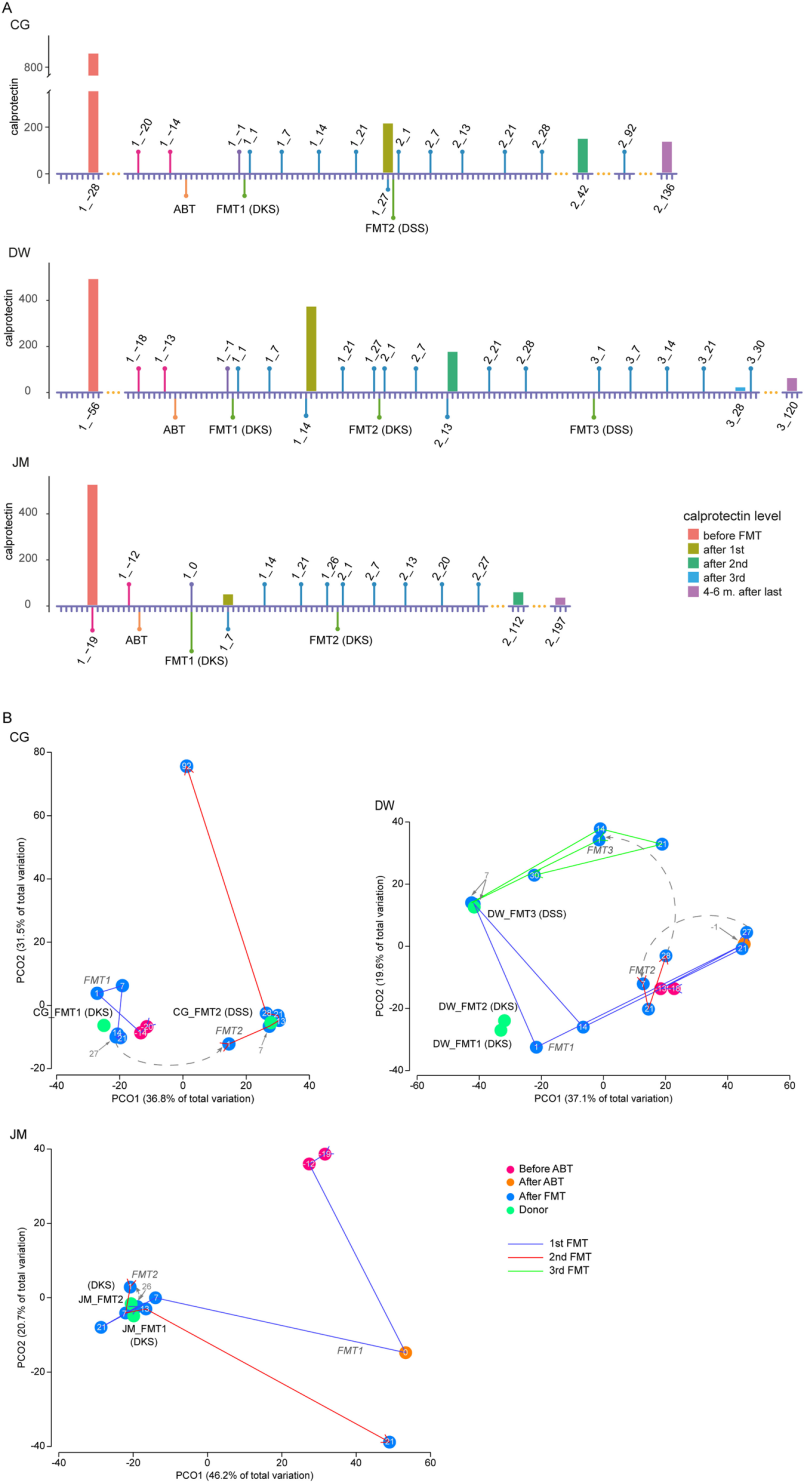
FMT treatment timeline and PCoA visualization. **A** Timeline of treatment process with FC levels. **B** PCoA based on gene expression profile of each sample. In A, sampling time points are indicated with the following: < No. FMT > _ < days since this FMT > . ABT is the timepoint for the antibiotic treatment. FMT1/2/3 indicates the timepoint for the corresponding FMT, while DKS or DSS in the bracket shows the donor used for this FMT. The height of the bars indicates the FC level. In B, each dot represents a sample, and the number in the dot indicates the number of days since the latest FMT. A negative number indicates the sample was taken before the first FMT. The trajectory lines show the timepoints across different FMT treatments. The start of each FMT is indicated in italic label. The donor sample used by each FMT is marked and colored in green, and the samples before treatment are in red, before FMT after ABT in orange, and after FMT in blue. To be able to compute the distance metric and visualize the data, only the top 200,000 expressed genes which account for 92% total mapped reads were taken into account

Before treatment, the patients showed elevated FC levels (849 μg/g for CG, 479 µg/g for DW, and 566 µg/g for JM (Fig. 2A, Table S1 sheet 1). The first FMT with the initial donor DKS resulted in a substantial drop of the FC levels for all patients except patient DW. However, the values remained at a high level for CG (*FC* = 219 μg/g). For patient DW, the reduction in FC level post-FMT1 was only 21% (decreasing from 479 to 377 μg/g). The second FMT for DW with the same donor reduced the FC level to around 200 μg/g. Then the FMT2 for CG and FMT3 for DW with a different donor (DSS) brought their FC levels to a normal range (150 μg/g for CG and 15 μg/g for DW). For patient JM, FMT1 was already successful and led to a low FC level (*FC* = 47 μg/g). The taxonomic composition (Fig. 1, Figure S2) and gene expression profile (Fig. 2B) of the active community showed a patient specific pattern. The initial communities of the patients were different from each other, and they responded to the treatment distinctly (Figs. 1 and 2B, and Figure S2). The active genera were *Prevotella*, *Ruminococcus*, or *Bifidobacterium* before FMT in pouchitis patients, while *Bacteroides*, *Faecalibacterium*, *Ruminococcus*, or *Prevotella* were active in donors. After FMT, significant engraftment could be observed for *Bacteroides*, *Faecalibacterium*, and *Prevotella*.

Principal coordinates analysis (PCoA) based on gene expression profile showed the temporal dynamics of the community before and during FMT. The gut metatranscriptomes of patient CG were initially close to those of the donor DKS before FMT (Fig. 2B). After FMT1 provided by DKS, they were even more similar to this donor accompanied by a substantial drop in the FC level. However, since the decreased FC level remained above 200 μg/g, a second FMT with a different donor (DSS) was performed. This FMT2 further reduced the inflammation of this patient according to the FC level, and the patients metatranscriptome now resembled that of the donor DSS. The sample 92 days after FMT2 showed an activity pattern which was distinct from both donor samples and the patient samples prior to treatment. Patient DW showed highly variable microbiota (Fig. 2B). The first and second FMT caused large fluctuations around the diseased state (before ABT) as well as towards and away from the donor communities. Specifically, FMT1 failed to trigger a significant reduction in the FC level, which remained much higher than 200 μg/g on day 15 post-FMT1. Moreover, between days 14 and 21 post-FMT1, the activity profile noticeably diverged from the donor cluster. After the second FMT with the same donor, the activity profile still deviated from that of the donor. By contrast, the profiles were much more stable and closer to the donor after the third FMT, where a different donor (DSS) was used. One month after the third FMT, FC dropped to a very low level and remained low for 4 months after FMT3. For this patient, the data show that the choice of the donor for FMT might have had a large influence on the quality of engraftment.

Remarkably, for patient JM, the first FMT changed the community which now resembled that of the donor and was maintained in that state for 1 month and already brought the FC measurement to a very low level (Fig. 2). Since the patient responded to the donor’s microbiota well, the same donor (DKS) was used again for the second FMT. After the second FMT, the community stayed similar to the donor state for 20 days but eventually shifted to a very different state. The final metatranscriptome sample, 27 days after the second FMT, differed from all previous community states and was closest to the community after ABT. However, the FC levels remained very low 4.5 and 6.5 months after the second FMT (*FC* = 56 and 32, respectively) which represent a normal status. Patient JM was the youngest recipient, and its pouch was only 16 months old, which might have helped remission to occur. Interestingly, human cytomegalovirus (HCMV) transcripts were found to be highly abundant in the samples from patient JM before FMT2 and in the sample JM_FMT1/2 from donor DKS (Table S1 sheet 6, Figure S5). The virus transcripts were also found in another sample DW_FMT1 from this donor used for the patient DW where no *Cytomegalovirus* was subsequently detected after this FMT, which may indicate an impaired immune system of patient JM and a potential problem with donor DKS.

**Fig. 3:**
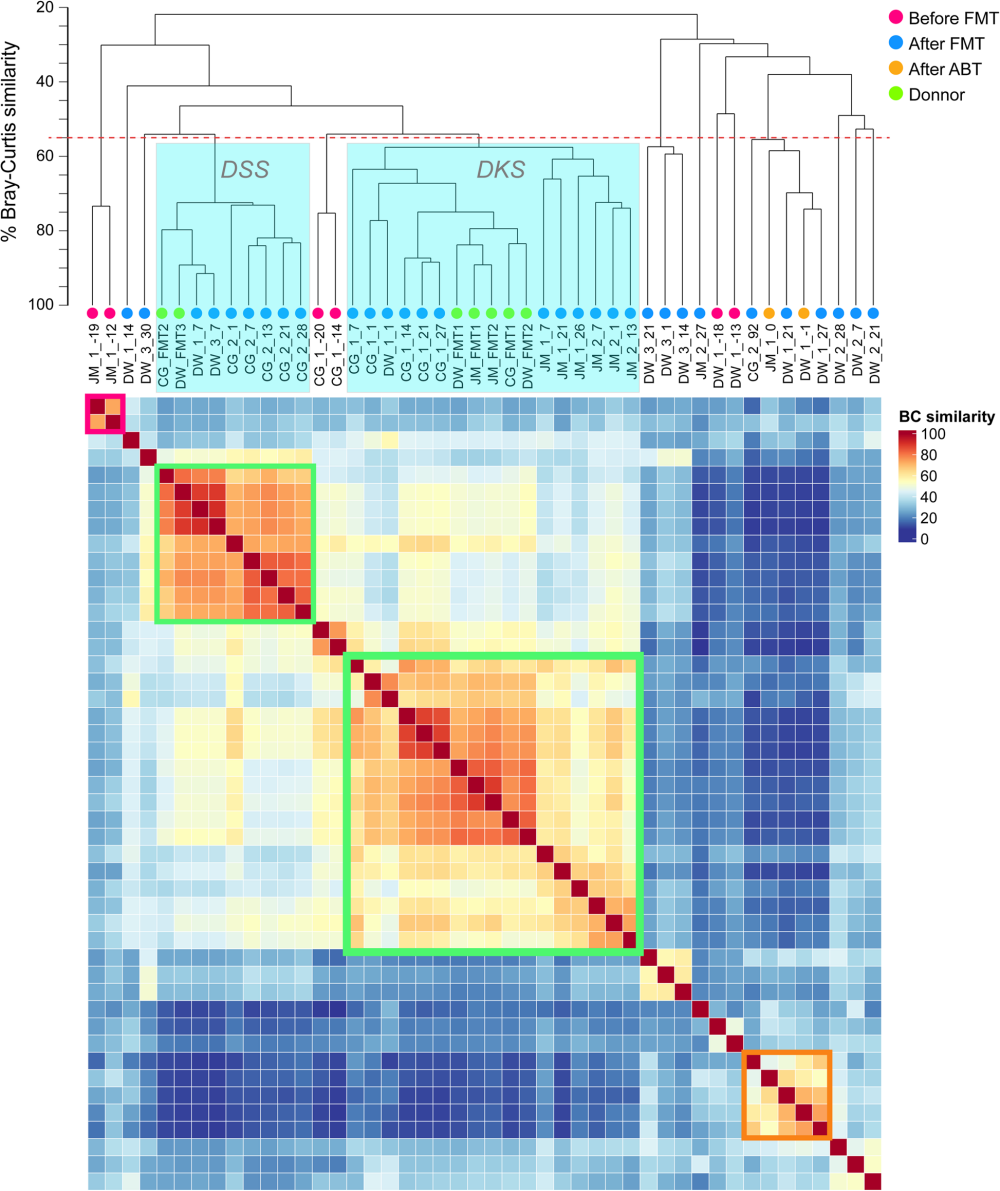
The clustering of the samples based on gene expression. Bray–Curtis dissimilarity was used to calculate the distance of the samples. The dotted red line indicates the similarity (100% - dissimilarity) cutoff of 55%. This cutoff clearly grouped the recipient samples after FMT into two clusters represented by corresponding donors. The heatmap below shows the similarity between each pair of samples, and it also demonstrates these two clusters apparently. The top 200,000 expressed were used in this visualization as in Fig. 2B

A clustering analysis of all communities (Fig. 3) showed that after FMT, the recipients’ communities were clearly clustered together with their donors into two groups (Fig. 3, Figure S6). The donor DSS, which was used for FMT2 in patient CG and FMT3 in patient DW, clustered together with the samples from these patients after FMT with one exception of 7 days after FMT1 for DW. Likewise, the donor DKS, which was used for all other FMTs, clustered together with the patient’s samples after FMT. All the communities in the cluster shared 55% gene expression profile similarity with each other. Smaller clusters were observed for the communities before treatment and after ABT.

### Activity changes between health and pouchitis

To discover the activity shifts of the communities and the microbial members that drove them, we compared the donor metatranscriptomes (five samples from donor DKS and two samples from donor DSS) to patient metatranscriptomes before treatment (two samples per patient). Using ALDEX2, we identified 6832 out of 815,055 genes to be differentially expressed (DE) between health and pouchitis (Table S1 sheet 7). More than 98% of those DE genes had a median effect size > 1.5, ensuring the statistical power of the analysis. Almost all of them were upregulated in health, while only 46 genes were upregulated in pouchitis. Among those DE genes, 658 were from *Bacteroides*, 407 from *Bifidobacterium*, 394 from *Alistipes*, 338 from *Blautia*, and 311 from *Faecalibacterium.* On the species level, 330 genes were differentially expressed by *Bifidobacterium adolescentis*, 90 by *Roseburia intestinalis*, 90 by *Eggerthella lenta*, and 66 by *Faecalibacterium prausnitzii* (Table S1 sheet 7).

**Fig. 4:**
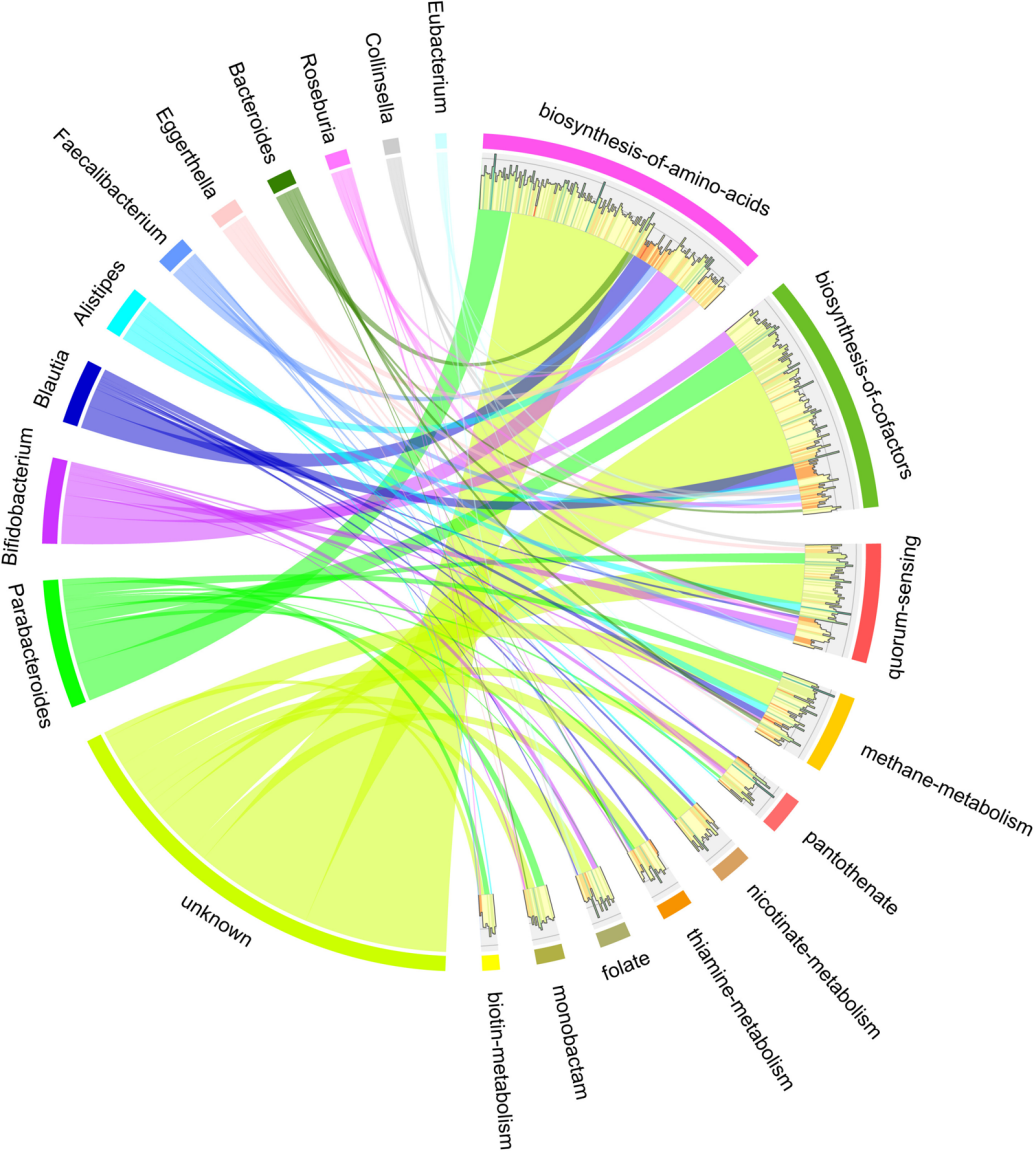
The microbial players driving the activity alterations in pouchitis. Circos plot showing KEGG pathways that were significantly enriched in healthy donors compared to pouchitis patients before medical intervention and the microbial taxa contributing to these pathways. The arcs on the left represent the microbial taxa grouped to genus level, and arcs on the right show the pathways. The width of the links between them indicates the number of differentially expressed KEGG ortholog (KO) genes from each taxon for a given pathway, and the color of the link matches the color of the microbial genus. The histogram below the pathway arcs shows the log2FC of the median of KO gene expression between health and pouchitis. For each pathway, only differentially regulated genes are shown. “Unknown” refers to differentially expressed genes that could not be assigned to a microbial genus

Gene-set enrichment analysis of 6,832 DE genes using ClusterProfiler [26] identified 65 KEGG pathways and 24 KEGG modules which were significantly differentially regulated between health and pouchitis (Table S1 sheet 8, Figure S7A). The most significant KEGG pathway enrichments were amino acid biosynthesis, co-factor biosynthesis, flagellar assembly, ribosome, carbon metabolism, vitamin B complex biosynthesis/metabolism (biotin, folate, nicotinate, pantothenate, and thiamin), selenocompound metabolism, sulfur metabolism, and bacterial chemotaxis (Figure S7, Table S1 sheet 8). The most significantly enriched KEGG modules in health were the biosynthesis of amino acids, fatty acids, heme, and vitamin B12 (cobalamin) (Figure S8A, Table S1 sheet 8). The data suggest that patient gut microbiota before treatment were unable to produce essential nutrients by themselves. By deconvoluting the pathways and modules into genus specific activity, we identified the genera driving those activity alterations. Figure 4 shows the contributing taxa to 10 out of 65 differentially regulated KEGG pathways, including biosynthesis of amino acids, biosynthesis of cofactors, quorum sensing, methane metabolism, pantothenate biosynthesis, thiamine metabolism, nicotinate metabolism, folate biosynthesis, monobactam biosynthesis, and biotin metabolism. The contributing genera were *Parabacteroides*, *Bifidobacterium*, *Blautia*, *Alistipes*, *Faecalibacterium*, *Eggerthella*, *Bacteroides*, *Roseburia*, *Collinsella*, and *Eubacterium*. Except for *Eggerthella*, they were all among the 25 most active genera in the stool microbiota of patients and donors (Fig. 1). Each of those genera contributed to several KEGG pathways, but to a different extent. For example, *Parabacteroides* drove the alteration of amino acid biosynthesis activity most strongly but was also a major player for differential regulation of the other pathways analyzed here. *Collinsella*, on the other hand, had a large role for quorum-sensing-related genes but a minor role for all the other pathways. From the perspective of the functional modules, their differential expression was usually driven by several genera. For example, vitamin-related pathways were differentially expressed by at least three genera, e.g., *Bifidobacterium*, *Parabacteroides*, and *Collinsella* for folate biosynthesis.

As we had a relatively small sample size, we analyzed a larger, independent dataset [18] from the HMP2 (https://www.ibdmdb.org/) to consolidate our findings. There are no published metatranscriptome datasets for pouchitis. This HMP2 dataset contains metatranscriptomes from UC patients (*n* = 53) and individuals without UC (*n* = 53). While the microbiome of patients with UC and pouchitis might differ due to modified anatomy after IPAA, the foundational pathology of pouchitis is rooted in UC [27, 28]. Thus, their microbiomes, while distinct, should share more similarities with each other than with a healthy state. In total, 5128 genes were differentially expressed between non-UC and UC, of which 4495 genes were upregulated in non-UC, while only 633 were downregulated. Genus *Alistipes* contributed to most of the upregulated genes (3240), followed by *Bacteroides* (110), *Faecalibacterium* (29), *Ruminococcus* (27), *Barnesiella* (26), and *Clostridium* (24). KEGG pathways and modules including biosynthesis of cofactors, biosynthesis of amino acids, ribosome, fatty acid biosynthesis, tryptophan biosynthesis, various vitamin B complex (folate, biotin, pantothenate, nicotinate, thiamine, riboflavin, pyridoxine) biosynthesis, and metabolism were upregulated in non-UC (Table S1 sheet 9, Figure S7B, Figure S8B). *Alistipes*, *Bacteroides*, and *Ruminococcus* were the main contributors to the upregulation of these pathways in the community. The activity alterations between non-UC and UC communities were highly similar to the changes that we found in our pouchitis dataset. In fact, 81% of the enriched KEGG pathways and 54% of the enriched KEGG modules were shared between the analyses of the pouchitis and UC metatranscriptomes (Figures S7  and 8 ). Interestingly, the microbial members driving those activity changes were very different. This analysis confirms the soundness of our results and allowed us to do more in depth analyses.

### Gene expression changes in synthesis of butyrate, B vitamins and metabolism of bile acids, and tryptophan

Since KEGG pathways and modules are rather broad categories, we manually analyzed functions known to be important for health in the gut microbiota. These include pathways for the biosynthesis of butyrate, vitamin B12 (cobalamin), vitamin B6 (pyridoxine), and metabolism of bile acids, tryptophan in more depth. Among them, B12 biosynthesis had already been identified as a significantly upregulated function in the KEGG module enrichment analysis described above.

We identified 1799 expressed butyrate biosynthesis genes in the total metatranscriptome, belonging to 28 ortholog butyrate biosynthesis genes that could be assigned to four different pathways (Figure S9). Among those expressed butyrate biosynthesis genes, 34 were differentially expressed between health and pouchitis, and they belong to nine ortholog genes (Figure S10, Table S1 sheet 10). Notably, all of them were massively downregulated in pouchitis with an average fold change of more than 100. Those DE butyrate biosynthesis genes were from *Blautia*, *Eubacterium*, *Faecalibacterium*, and *Roseburia* (Figure S10, Table S1 sheet 10). Using Fisher’s exact test, we found that the butyrate biosynthesis genes were strongly enriched among all differentially expressed genes (*p* = 2.123e-05, enrichment ratio 2.285 (95% *CI*: 1.576, 3.209).

Bile acids metabolism was represented through 638 genes which were expressed in the communities. Among them, ten genes were differentially regulated between health and pouchitis (Figure S11, Table S1 sheet 11). They were downregulated in patients before treatment with an average fold change over 150. Interestingly, only orthologues of *cbh* and *baiN* were comprised. There was no differential regulation of any of the genes involved in the formation of secondary bile acids (*baiA-I*). The DE *cbh* genes originated mainly from *Alistipes* and *Collinsella*, whereas *baiN* originated from *Blautia* and *Faecalibacterium.*

For vitamin B12 biosynthesis, we identified 4874 expressed genes. Of those, 40 were differentially expressed between health and pouchitis, and all of them were upregulated in health with an average fold change around 200 (Figure S12, Table S1 sheet 12). The genera contributing most transcripts to vitamin B12 biosynthesis were *Alistipes*, *Anaerobutyricum*, *Bifidobacterium*, *Blautia*, and *Parabacteroides*.

For vitamin B6 biosynthesis, we identified 1249 expressed genes in the total metatranscriptome, of which 11 genes were differentially regulated between health and pouchitis, and all of them were upregulated in health with an average fold change above 200 (Figure S13, Table S1 sheet 13). Those differentially regulated genes were mainly derived from *Alistipes*, *Bifidobacterium*, and *Blautia*.

Tryptophan metabolism was represented by 1168 expressed genes in the dataset, of which 16 genes were differentially expressed. However, only tryptophanase of *Alistipes*, a documented producer of indole from tryptophan can be assumed to be crucial for tryptophan metabolism (Figure S14, Table S1 sheet 14) [29].

### Engraftment of essential functions during FMT

Next, we asked how these 6832 DE genes between donors and pouchitis (see above) were expressed in patients over the complete FMT treatment. The heatmap of Figure S15 shows the expression of those genes clustered according to genus in all samples, and the barplot on the top summarizes the relative expression in all genera. The short-term engraftment of the DE genes in all patients after FMT can clearly be seen, as well as their loss 4 weeks after FMT (barplot on the top of Figure S15). An exception was the second FMT for patient DW, where the activity of the donor microbiota was not substantially engrafted in the patient. A third FMT for this patient using a different donor indeed resulted in clear engraftment of the activity represented by the DE genes for a short time.

**Fig. 5:**
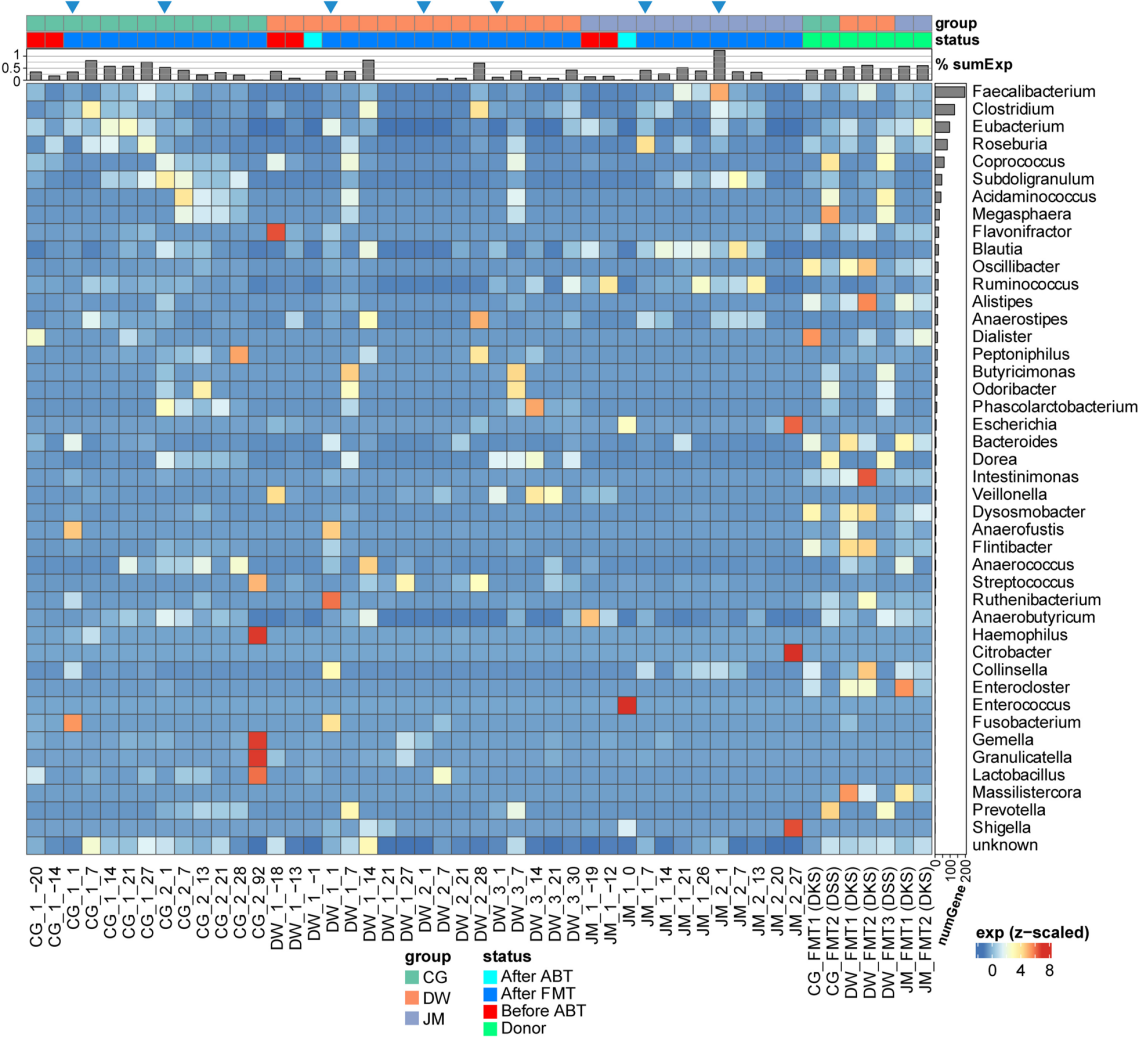
Expression of butyrate synthesis genes during FMT in different taxa. Each cell in the heatmap displays the Z-score transformed relative expression of butyrate synthesis genes within a specific genus for a given sample. The barplot on the top shows the sum of the relative expression of all butyrate synthesis genes for each sample. The right bar illustrates the total number of expressed butyrate biosynthesis genes for this genus across all samples. The genera are sorted based on the number of contributing genes, from the most to the least. The blue triangle on the top of column annotation bar indicates the first time point after each FMT. The column annotation bars are the same as in Fig. 1

We then analyzed the expression of DE genes in particular functions including synthesis of butyrate (Figure S10), metabolism of bile acids (Figure S12), synthesis of vitamins B12 (Figure S12) and B6 (Figure S13), and metabolism of tryptophan (Figure S14) (see above) in the samples from the patients during FMT. The expression levels of these genes were temporarily restored by the FMT but got lost again at the later time points. Particularly, the overall expression level of all butyrate biosynthesis genes also showed a clear engraftment (Fig. 5). The most important genus expressing butyrate biosynthesis genes was *Faecalibacterium* followed by genera *Clostridium*, *Eubacterium*, and *Ruminococcus* (Fig. 5, right-side barplot). After each FMT (except FMT2 for patient DW), the expression of those butyrate biosynthesis genes increased and then went back to a low expression level around 1 month after the FMT (Fig. 5). Notably, the first FMT for DW led to only a transient increase in the expression of those genes (Fig. 2A). The FMT2 resulted in increased expression of butyrate synthesis genes, but the overall metatranscriptome profile still diverged from the donors, and the FC level persisted at a high value. In contrast, the third FMT with a different donor effectively elevated the expression of butyrate synthesis genes for 1 month, accompanied by a significant reduction in FC concentration. The overall expression levels of all genes involved in tryptophan metabolism had similar patterns as the butyrate biosynthesis genes, except that the last sample of patient JM had a high expression level of tryptophan metabolism genes (Figure S16). Among those genes, only a DE gene encoding tryptophanase (*tnaA*) was crucial for tryptophan metabolism and engrafted in CG after the second FMT (CG_2_1) and in DW after the third FMT (DW_3_7) (Figure S14, Table S1 sheet 14). The top genus contributing to the alteration of metabolism of tryptophan was *Alistipes*. However, the overall expression of genes involved in the metabolism of bile acids, and biosynthesis of vitamins B12 and B6, did not show such a pattern during FMT.

### Activity of Faecalibacterium prausnitzii during FMT

**Fig. 6:**
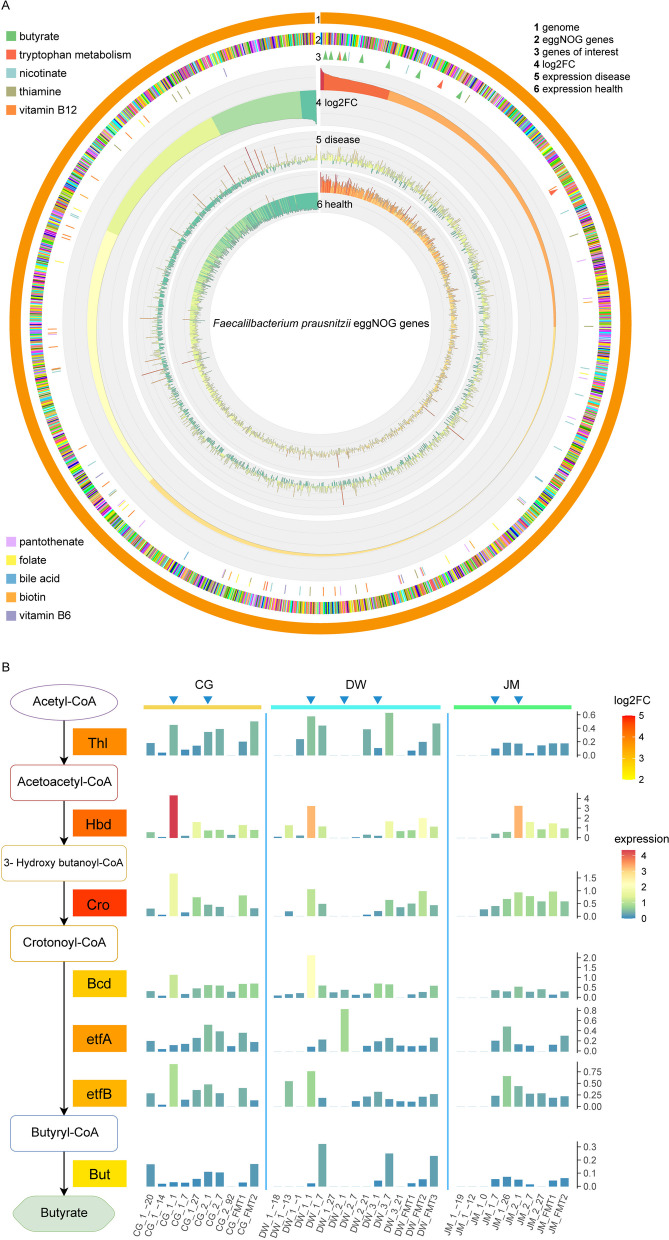
Activity alterations of *Faecalibacterium prausnitzii* during FMT. **A** Gene expression profiles of *F. prausnitzii* in health and disease (eggNOG genes). **B** The gene expression changes of acetyl-CoA pathway for butyrate biosynthesis in *Faecalibacterium prausnitzii*. In A, the circos plot is based on all expressed genes assigned to *F. prausnitzii* which were then grouped to eggNOG genes according to the ortholog annotation. Rings show (from outside to inside) the following: #2 all eggNOG genes; #3 butyrate synthesis genes (green arrow), genes involved in tryptophan metabolism (red arrow), the other genes that are involved in secondary bile acids metabolism, vitamins B1 (thiamine), B3 (nicotinate), B5 (pantothenate), B6 (pyridoxine), B7 (biotin), B9 (folate), and B12 (cobalamin) biosynthesis, are marked with bars in distinct colors; #4 log2 fold change of gene expression between health (donors) and pouchitis (patients before treatment); #5 expression level of genes in pouchitis (patients before treatment); and #6 expression level of genes in health (donors). For B, only patient’s samples before FMT, 1 week, and the last timepoint after each FMT were included. The colored box with gene names in the pathway shows the log2FC between donors and pouchitis. Red indicates the strongest upregulation in donor in comparison to pouchitis. The barplot illustrates the expression level of each gene during the treatment process. The height and color of the bar indicate the relative expression level (number of reads from that gene normalized by number of total reads from *F. prausnitzii*) of the gene. The blue arrow at the top of the barplot marks the first timepoint after each FMT

Since *F. prausnitzii* was shown to be the most active microbe contributing to the alterations of butyrate biosynthesis activity between health and pouchitis and because of its documented importance for a healthy gut, we analyzed its activity in more depth. As it was shown above, the overall abundance of transcripts from *F. prausnitzii* was higher in donors than in pouchitis, and it was especially high in donor DKS (Figure S2). We then extracted all the reads belonging to *F. prausnitzii* and grouped them to 2154 eggNOG genes and 20 Clusters of Orthologous Groups (COG) categories. EggNOG is a hierarchical, functionally and phylogenetically annotated orthology resource [30], and the IGC reference gene database contains eggNOG annotation as well as COG categories. The COG category composition did not demonstrate a clear pattern across the samples (Figure S17). We then analyzed the expression of eggNOG genes of *F*. *prausnitzii* represented by the median of centered log-ratio (CLR) transformation for the read counts (Fig. 6A). Due to the fact that transcriptome sequencing data are compositional, and the sequencing depth is variable across samples, we could not simply normalize the read counts of genes with cell numbers to reflect the true mRNA/cell values [31]. Therefore, we need a library size normalization method which accounts for the compositional property of transcriptome data. CLR transformation is formally like effective library size normalization and delivers meaningful values for abundance and expression comparison [32, 33]. The median of the CLR values of a given gene in donors and pouchitis was used to determine the fold change of the expression of that gene. The results showed that *F. prausnitzii* had different activities in donor and pouchitis. The most strongly upregulated genes in donor were transporters and membrane proteins, and the most downregulated genes were involved in nucleotide metabolism, amino acid metabolism, capsular polysaccharide biosynthesis, toxin-antitoxin system, and restriction modification system. There were eight eggNOG genes involved in butyrate biosynthesis in total, and they were all higher expressed with seven to 27-fold in terms of the median CLR value in donor (Table S1 sheet 15). Mapping the individual genes back to pathways suggested that butyrate biosynthesis was mainly contributed by acetyl-CoA pathway. All genes involved in the acetyl-CoA pathway were expressed in *F. prausnitzii*, and they were upregulated in donor compared to pouchitis (Fig. 6B). Gene *Thl* and *But* were much higher expressed in donor DSS compared to donor DKS, while *Cro* gene was much more active in DKS. One to 7 days after FMT, the relative expression levels of the acetyl-CoA pathway genes were increased drastically in patient’s microbiome (Fig. 6B). We observed that when *F. prausnitzii* was less active in terms of the total transcript abundance, its butyrate biosynthesis genes tended to be much lower expressed relative to all other genes. This might be because *F. prausnitzii* needs to produce butyrate to thrive. In the HMP2 UC and non-UC dataset, *F. prausnitzii* was not as active as that in our FMT donor samples, and no substantial differences of the butyrate biosynthesis gene expression between non-UC and UC were observed.

## Methods

### Sample collection

Stool samples were obtained during a clinical study on FMT for patients suffering from antibiotic-resistant pouchitis [14]. We followed the metatranscriptomes during the whole treatment process from three patients that experienced clinical remission and had a complete record. Clinical data and FMT treatment results from these patients CG, DW, and JM were initially reported in [14]. It is important to note that patient DW underwent three FMTs, while both CG and JM received two. The 2016 publication only describes the first of the two FMTs received by patient JM. Additionally, in contrast to the data presented in the 2016 table, patient CG underwent two FMTs instead of the stated three. The patient samples were named with a format as follows: < patient ID > _ < no. FMT > _ < days since this FMT > . Donor samples were named as follows: < recipient patient ID > _FMT < no. FMT > . The FC levels were measured before the treatment and after each FMT, as well as 4–6 months after the last FMT to confirm the long-term efficacy of the treatment. A calprotectin level above 200 μg/g indicates an inflammatory condition of the patient. Therefore, an improvement in the patient’s condition is defined by a substantial drop in the FC level from well above 200 μg/g to a level below this threshold. The final remission after the last FMT was characterized by a PDAI score below seven (Table S1 sheet 1).

### RNA extraction and sequencing

RNA was extracted according to [34] using the PowerMicrobiome RNA Isolation Kit (MoBio). A Ribo-Zero Gold rRNA Removal Kit (Epidemiology) (Illumina, USA) was then used for rRNA depletion with ethanol precipitation according to the manufacturer’s instructions. Paired-end mRNA Illumina sequencing libraries were created by a ScriptSeq kit (Illumina). The HiSeq 2500 sequencer platform was used to generate strand-specific 2 × 110 bp paired-end sequencing reads.

### Quality control and preprocessing of the sequencing reads

Primers and sequencing adapters were trimmed from raw sequencing data, followed by clipping the low-quality bases with score < 20 from 5′ and 3′ ends of the reads using Fastq-Mcf (https://github.com/ExpressionAnalysis/ea-utils) [35]. After clipping, the reads containing less than 50 nucleotides were removed. rRNA reads were eliminated using SortMeRNA v2.0 [36] with the default parameter settings. The human reads were removed by assigning reads against human reference using Kraken.

### Reference database construction

The IGC database provides a comprehensive gene catalog for the human gut microbiota [23]. This nonredundant gene set has been functionally and taxonomically annotated which provides a great overview of the taxonomic and functional diversity of the human gut microbiota with high resolution. Moreover, this database allows us to profile the taxonomy and function of human gut metatranscriptomic data. Using IGC as reference database, we could assign 86.71% of all putative microbial mRNA reads with BWA-MEM [24]. To further improve the mapping rate, a reference gene catalog named IGC59G was constructed. The pipeline for the construction of IGC59G is depicted in Figure S1A. Before mapping the reads against IGC, the quality-controlled reads were taxonomically assigned using Kraken onto a database that we called Kraken-BVAHPF which contains all bacteria, archaea, viruses, plasmids, fungi, and human genomes from NCBI RefSeq database. By using Kraken, only 43.82% of all quality-controlled reads could be classified, of which 3.78% originated from the human hosts and were removed. Interestingly, among the reads which were not mapped to the IGC database, 54.33% could be classified by Kraken using Kraken-BVAHPF. Therefore, a more comprehensive database was built based on the IGC database and the Kraken database. To do so, in the Kraken database, the representative genomes of the species which had more than 100,000 IGC unmapped reads assigned in any sample were extracted. In total, there were 59 genomes. The coding sequences (CDS) of those 59 genomes were then clustered with an identity at 0.95 and coverage at 0.9 using Roary [37] and CD-hit [38]. The resulting nonredundant genes were mapped against ICG genes using BLASTN to exclude the redundant genes from 59 genomes. The genes mapped onto any of the sequence in ICG with an identity > 95% and coverage > 90% were eliminated. Finally, in total, 60,658 unmapped genes from those 59 genomes were added into the ICG database to constitute the final gene catalog IGC59G. Using this improved database and short-read aligner BWA-MEM, 87.91% of nonhuman reads could be assigned. This represents an overall improvement by 1.2% compared with the original IGC database, but in some samples, the improvement was much larger, particularly for CG_2_92, DW_1_-1, DW_1_27 which had an increase of the alignment rate over 10%.

### Taxonomy assignment and functional annotation of reference genes

Since the annotation of the IGC database was several years ago, the NCBI RefSeq database has been expanding. Moreover, the original taxonomy information of IGC does not include species level. Therefore, we performed a taxonomy assignment for the IGC59G database using centrifuge [25]. Human, bacteria, archaea, viruses, and fungi genomes were downloaded from NCBI RefSeq on March 18, 2020, and used for updating the missing taxonomy information and assigning the species for each reference gene sequence. Centrifuge can be used to assign taxonomy information with high accuracy for both short reads and long sequences. Hits with score >  = 200 and hitLength/queryLength >  = 0.2 were taken into account. In order to investigate the expression of butyrate biosynthesis genes, the reference genes were annotated using DIAMOND [39] (parameters: –id 60 -e 1e-9 –query-cover 70). The sequences and annotation information of butyrate biosynthesis genes were downloaded from a previous work [40] that collected 2766 butyrate biosynthesis genes from 149 species derived from the human microbiome. Those genes belong to 28 ortholog genes and four pathways. The eggNOG, KEGG annotation for the additional genes in IGC59G was performed using DIAMOND based on the eggNOG and KEGG protein databases with the same criteria utilized for butyrate biosynthesis gene annotation.

To compute the gene expression profile, the nonhuman reads were mapped against the reference gene database. The taxonomic composition of the metatranscriptome was determined based on the taxonomy information of each gene in the reference database (Figure S1B). PRIMER-ANOVA + was employed to perform the analyses of dominance, diversity, Bray–Curtis similarity, and PCoA [41] (Figure S1B). Sample DW_2_1, JM_1_14 and JM_2_20 were excluded from the PCoA since they had less than 10 million mapped reads. The communities with Bray–Curtis similarity above 55% were considered as similar.

### Calculation of differential expression and KEGG pathway analysis

To identify differentially expressed genes between donors and pouchitis, a differential expression (DE) analysis was performed using ALDEX2 based on the read counts. ALDEX2 [42] is an ANOVA-like tool for high-throughput sequencing data including transcriptome and metatranscriptome data. ALDEX2 performs statistical test on CLR transformed read count to identify differentially expressed genes between conditions. Only genes which had an average read count above one were considered. The R package edgeR [43] was additionally used for DE analysis to confirm the robustness of results computed by ALDEX2. Both ALDEX2 and edgeR are well-suited for analyses involving data sets of relatively modest sample sizes. Interestingly, edgeR detected ten times more DE genes compared with ALDEX2, while ~ 90% of DE genes predicted by ALDEX2 were also identified by edgeR. Therefore, in the downstream analysis, we used the results from ALDEX2 which are apparently more rigorous. For our FMT dataset, the genes with FDR <  = 0.05 (false discovery rate: Benjamini–Hochberg corrected *p*-value of Wilcoxon test) were considered as significantly differentially expressed. Since the HMP2 dataset has much larger sample size, we used a more stringent FDR cutoff of 0.01. The R package ClusterProfiler was used for KEGG pathway enrichment analysis. Only pathways or modules with FDR <  = 0.05 were considered as significantly enriched. To analyze the eggNOG gene expression of *F. prausnitzii* in donors and pouchitis (Fig. 6A), we performed a CLR transformation on the read counts as follows:

$$\mathrm{CLR}(x)=\left(\mathrm{log}\left(\frac{{x}_{1}}{g(x)}\right),\dots ,\mathrm{log}\left(\frac{{x}_{n}}{g(x)}\right)\right)$$

Where ${x}_{i}$ is the read count of gene $i$ and $g(x)$ is the geometric mean of all genes.

## Discussion

The gut microbiome of healthy humans is stable in the long term, responds rapidly but usually transiently to changes in environmental conditions (e.g., food, life style, medical intervention) [44], and is so characteristic that it can be used to fingerprint the host [45]. Pathogenic conditions result in characteristic shifts in relative abundance of taxa [46]. Maintaining a healthy gut microbiota might therefore prevent or even cure various diseases and malfunctions, including obesity, chronic inflammatory bowel disease, rheumatoid arthritis, colorectal cancer, and neurological diseases [47, 48]. Modifying the gut microbiome composition by dietary intervention, probiotics, or FMT is therefore of huge interest. In the case of FMT, a highly diverse, complex microbiota is transferred into a host who at the time of intervention carries a dysbiotic gut microbial community which nonetheless might have developed into a stable, resilient state [44, 49].

Here, we provide the first time-resolved longitudinal case study of stool microbiota of healthy donors and pouchitis patients before and during FMT using metatranscriptome analysis. For this clinical indication, large cohort studies are not yet available, with only a handful of clinical trials currently underway. Our study first compared the metatranscriptomes of pre-FMT patients and healthy donors. We then tailored our examination on post-FMT activity alterations, focusing on each patient individually due to their distinct responses to treatment. This approach accurately captured the unique patient responses to FMTs, meanwhile characterized common patterns and trends across all patients. In contrast to metagenome studies, we observed the actual activity of the microorganisms, rather than their genetic potential. Metatranscriptomes not only close the gap between genomic potential and actual activity but also they allow to focus on key genes and functions and the underlying microbial players. For example, in colorectal cancer, the tumor promoting roles of colibactin-producing *E. coli*, enterotoxigenic *Bacteroides fragilis*, and *Fusobacterium nucleatum* can be pinned down to a handful of specific genes which are only found in some strains of the species [50].

Our data show a striking engraftment of donor microbial activity in patients directly after FMT. PCoA and the clustering based on gene expression profile showed that patient microbiota activity directly after FMT was indistinguishable from that of the respective donor (except for DW after FMT2), i.e., they resembled donor DKS if that had provided the microbiota and donor DSS if that was used. Moreover, pathways or functional modules that were shown to be highly expressed in donors but weakly expressed in pouchitis patients before treatment (e.g., synthesis of butyrate, amino acids, cofactors, vitamin B complex) were highly expressed in the first 1 to 2 weeks after FMT. We found a notable association between FC levels and activity engraftment post-FMT. Specifically, higher FC levels tend to occur when the recipient’s activity profile diverges from the donors.

We identified the genera that drove the difference between health and pouchitis, namely *Parabacteroides*, *Bifidobacterium*, *Blautia*, *Alistipes*, *Faecalibacterium*, *Eggerthella*, *Bacteroides*, *Roseburia*, *Collinsella*, and *Eubacterium*. They were among the 25 most active genera in our metatranscriptomes, except for *Eggerthella*. *Eggerthella lenta* has been shown to be able to inactivate the cardiac drug digoxigenin in the gut by expressing a specific operon [51]. It also plays an important role in metabolizing lignin and plant-derived metabolites in the gut [52]. On the other hand, *E. lenta* is an emerging pathogen and can cause bloodstream infections [53, 54] and was correlated with rheumatoid arthritis [55]. Because the genus *Eggerthella* was an important driver of activity alterations between health and pouchitis, it might be a useful component in probiotics of defined composition. However, the selection of the species and the strain within the species are of utmost importance [56].

Our data show specific engraftment of donor microbiota directly after FMT, so we asked if DKS was a superior donor compared to DSS or the other way around. A study on FMT for patients with ulcerative colitis found that seven of the nine patients that entered remission had received FMT from the same donor [57], which was therefore assumed to be a super donor [58]. Our data show that two patients did not respond well to the FMT from donor DKS, but their FC levels decreased drastically after FMT with donor DSS. Another potential problem with DKS is that its community contained HCMV sequences. HCMV was also active in patient JM before the second FMT. HCMV is a double-stranded DNA virus, and most adults have been infected by HCMV in their life [59]. It remains latent in lymphocytes in the body throughout life with few genes being transcribed after initial infection and becomes reactive when the host’s immunity is suppressed. In the HCMV-positive samples from donor DKS and patient JM, all HCMV genes were active (Table S1 sheet 6) suggesting a suppressed immunity of the host at the sampling time.

*F. prausnitzii* is an obligate anaerobe from the Ruminococcaceae that comprises about 5% of the total healthy colon microbiota [60], ranging from 0.96% in newborns to 7.6% in adults [61]. Decreased abundance of *F. prausnitzii* has been found in ulcerative colitis [62] and Crohn’s disease [63]. *F. prausnitzii* is able to suppress inflammatory responses [64], and it produces the short-chain fatty acid butyrate which supports the integrity of the intestinal mucosa [65]. It is therefore a strong candidate for intervention by dietary modifications, probiotics, and fecal microbiota transfer (FMT) [66, 67]. *F. prausnitzii* was much more active in the healthy donors compared to pouchitis patients in terms of total transcript abundance, and it was especially active in donor DKS. Moreover, *F. prausnitzii* manifested different activity in health and pouchitis. The expression levels of eight eggNOG genes involved in butyrate synthesis mainly via the acetyl-CoA pathway were low in pouchitis and elevated immediately after each FMT. The clinical outcome represented by FC level seems to correlate with the engraftment of the butyrate synthesis activity. *F. prausnitzii* cannot thrive in the presence of oxygen since it is an obligate anaerobe [68]. Large changes in ambient oxygen levels along the intestine have a major influence on the survival of obligate anaerobes and their fermentation ability [69]. Increased oxygen levels in the gut have been suggested to cause dysbiosis typical of inflammatory bowel disease [70, 71].

In health, genes including *baiN* and *cbh* which were previously assumed to be involved in secondary bile acids formation were upregulated in *Blautia*, *Faecalibacterium*, *Alistipes*, and *Collinsella*. In fact, the enzymes involved in the formation of secondary bile acids have only recently been identified [72], and in contrast to previous assumptions, *baiN* identified in *Lachnoclostridium scindens* as encoding a 3-dehydro-bile acid Δ(4,6)-reductase [73] is obviously not involved in the formation of secondary bile acids. Accordingly, various bacteria have been observed to encode genes similar to *baiN* without the presence of *baiA-I* genes indicating a different *baiN* function. Previous analysis of the bile acids profile in inflammatory bowel disease has observed a significant increase in the level of conjugated bile acids compared to healthy individuals indicating a lowered deconjugation activity in disease [74] which is consistent with the lowered *cbh* expression observed here in pouchitis.

Remission required repeated FMTs. At the clinical endpoint, the engraftment of donor communities differed among the three patients. In patient DW, the community activity profile at the last sampling point was similar to that of the healthy donor. In the other two patients, which were both described as being in stable remission [14], the final communities differed from the pre-treatment condition as well as from the donor communities. Similar findings were reported in an analysis of long-term engraftment of FMT capsules as therapy for CDI, where all 18 patients were in stable remission, but engraftment of donor microbiota was only observed in 61% [75]. It is possible that the stool microbiota of the patients were still not stable [76]. Since we had no sample from the patients back in the healthy state, we could not determine if their stool community activity was evolving towards their original individual state. Remission could restore also the eating habits of the patients, which may alter their gut microbiome [77].

In the long run, probiotics consisting of well-characterized strains should be used rather than FMT to avoid the costly screening of donors and the risk of transferring unwanted genetic traits. The findings from our study can inform the design of probiotics. A healthy stool microbiota is characterized by high diversity and owes its resilience to the fact that the synthesis of essential functions is not delegated to a single species, and thus at risk, but provided by a consortium of species with different adaptations and ecological niches (insurance theory) [3, 18, 44, 49]. Accordingly, when we analyzed the contribution of the abundant microbial taxa to the activity alterations of KEGG pathways in pouchitis compared to health, we found that multiple genera contributed to each of the activity alterations investigated. The traditional method of searching for a single species expressing the desired trait most strongly under laboratory conditions does not account for this complexity. Providing multispecies probiotics with large Shannon diversity rather than high-performance single strains might increase the chance of engraftment.

Because of our small sample size, we sought additional evidence regarding activity alterations in pouchitis and related conditions. To this end, we compared the dysbiotic microbiome activity profiles of UC patients (*n* = 53) with those of healthy controls (*n* = 53) from the HMP2 project. Patients with pouchitis after IPAA surgery have an altered anatomy compared to UC patients and healthy individuals. However, the fundamental pathology of pouchitis is rooted in UC, often being considered as a “recurrence of UC” within the pouch ileal mucosa [27]. Despite some distinctions, pouchitis and UC exhibit substantial overlap in their clinical and pathological features [28]. This close pathological linkage suggests that the stool microbiomes of pouchitis and UC patients, while distinct, should share more similarities with each other than with those of healthy individuals. Interestingly, similar activity alterations that we observed for pouchitis patients was found in the HMP2 dataset for UC patients, but the main taxa that contributed to these changes were different. An increasing number of studies underscore the intricate relationship between the taxonomic composition, functional potential, and actual functional activity of microbial communities. An analysis of oral and stool microbiota from different subjects highlighted that metatranscriptional profiles are markedly individual specific, with their variability falling between that of DNA-level functional profiles and microbial composition [78]. Subsequent study delving into the gut microbiota of IBD patients noted a general alignment between functional potential and metatranscriptome expression yet revealed that certain taxa display a weak correlation between their DNA and RNA abundances [18]. Another work on human fecal microbiota delineated pathways into “core” and “variable” subsets based on their prevalence in metatranscriptomes, revealing that the “core” pathways are often transcribed by different microorganisms [79]. Our previous investigation on vaginal microbiota suggested that despite taxonomic variability, the overall activity profiles remained remarkably congruent across subjects in bacterial vaginosis [80]. We hypothesize that under similar environmental and host conditions, the communities can manifest similar expression patterns for “core” functions, even when their taxonomy structures differ. This phenomenon can be attributed to the inherent redundancy in the genetic potential of most microbial species. Schirmer and co-workers also revealed that disease-related changes in the gut environment can affect microbial expression across different organisms and pathways sometimes without altering functional potential [18]. Similarly, a mouse study observed that host genetic shifts could change gut microbial gene expression without significant taxonomic alterations [81]. This may be because microbial gene expression tends to adapt rapidly to host condition changes, often preceding alterations in functional potential and taxonomic structure. Therefore, the metatranscriptome offers a prompt and holistic insight into disease-associated microbial shifts, capturing signals that metagenomic analyses might overlook.

In our study, it was not realistic to obtain metatranscriptomes from control patients that underwent placebo FMT. However, we observed a correlation between metatranscriptome engraftment and clinical outcome for patient DW who was treated by stool preparations from two different donors. The initial FMT treatment (donor DKS) was not effective, as evidenced by the lack of a significant decrease in FC level (FC > 200 after FMT1, Fig. 2A). This lack of efficacy was corroborated by the metatranscriptome data, which showed diverging activity composition in the PCoA (Figs. 2B and  3) and lack of expression of butyrate biosynthesis genes (Fig. 5). Subsequent FMT treatment with the same donor also failed to align the patient’s activity profile with the donors. The patient was then treated by a different donor (DSS) which resulted in a very low FC level 4 weeks after FMT3 (Fig. 2A). The activity profile on day 30 post-FMT3 resembled that of the donor (Figs. 2B and 3), and butyrate biosynthesis gene expression was relatively high (Fig. 5). A recent study examining the efficacy of FMT treatment in CDI patients based on strain-resolved metagenomic analysis of stool microbiota underscored that successful FMT recipients displayed significantly higher donor strain engraftment than those with failed FMT [82]. Complementing this finding, our data for patient DW revealed a similar pattern of activity engraftment during FMT. These observations suggest that donor engraftment on the metatranscriptomic level might be important for the success of FMT. However, the same initial donor worked well for patient JM. Also, we cannot exclude factors other than the change of donors that caused the FMT success in patient DW.

As of 2019, only three clinical studies had examined the effectiveness of FMT [83]. While two reported either no notable improvements or only a mild alleviation of symptoms, Stallmach et al. [14] observed that 80% patients (4/5) experienced over 3 months remission after repeated endoscopic FMTs using different donors. Choice of donors, FMT method, and frequency of FMT are among the reasons discussed for these differences [12, 84]. The subsequent first randomized proof-of-concept study applied an initial endoscopic FMT followed by daily stool capsules from the same donor for 2 weeks, but only one of six patients achieved antibiotic-free clinical remission [11]. The second randomized double-blind study in 2021 found similar outcomes for the placebo FMTs and treatment FMTs [17]. Today, taking nine studies into account, no definite conclusion can yet be reached [85], but it is premature to dismiss FMT as a treatment for antibiotic-resistant pouchitis. FMT has proven robust for rCDI [86]. However, the etiologies of pouchitis and UC are much more complex, involving host genetics, environmental factors, the patient’s healthy microbiome, and—in the case of pouchitis—surgery [87]. And the dysbiosis in the microbiome is not well understood due to the reliance on the 16S rRNA marker gene, which provides limited functional information [88]. The key challenge may be identifying an “ideal” donor for pouchitis, or perhaps even an ideal donor for pouchitis in a specific patient, making this treatment an option for personalized medicine. The randomized clinical studies reported above understandably used a single donor to reduce complexity. Our study is the first to examine engraftment of different donors at the metatranscriptomic level in FMT, highlighting the importance of donor selection.

The findings in this study have been obtained using a rigorous statistical approach, but conclusions should be interpreted with caution due to the nature of a case study, namely a small sample size and many potentially confounding factors that could not be taken into account. However, they set a foundation for future larger cohort studies to enable a more comprehensive understanding of key microbial players and associated activities that contribute to clinical success in FMT for pouchitis.

## Conclusions

Our study demonstrated a clear although short-lived engraftment of the activity of donor gut microbiota in patients with pouchitis after each FMT. Activity changes for key functions like biosynthesis of amino acids, cofactors, B vitamins, and butyrate were driven by several taxa, of which some have already been shown to have probiotic potential. The engraftment of butyrate biosynthesis activity from *F. prausnitzii* was well correlated with the patient’s inflammation status. The results suggest that repeated FMTs trigger a shift of the pouchitis microbiota towards a healthy state. In the future, probiotics with high taxonomic diversity consisting of numerous well-characterized strains should be used rather than FMT to avoid the costly screening of donors and the risk of transferring unwanted genetic material.

### Supplementary Information

**Additional file 1: Fig. S1. **Reference database construction and metatranscriptome data analysis workflows. (A) The construction of the gene database based on IGC for function and taxonomy assignment. Kraken BVAHPF represents the Kraken database built from NCBI genomes of bacteria, viruses, archaea, human, plasmids and fungi. (B) The metatranscriptome data analysis workflow. **Fig. S2.** Taxonomic composition of the expressed genes (transcripts) on genus (left) and species (right) level for patient CG (A), DW (B) and JM (C). The top 12 most active genera for each patient are shown. Those genera contributed over 90% of taxonomically assigned reads. Relative abundance was normalized by the total number of mapped reads for each sample. The portion of the bar which was left blank represents genera with an average abundance <1% and unknown taxa. For the species level, only the top 17 species with highest average transcript abundance are shown. **Fig. S3.** Composition of communities of patients CG, DW and JM based on 16S rRNA gene sequencing (genus level). **Fig. S4.** Cumulative rank abundance curve on the level of genus for patient CG (A), DW (B), JM (C) and their respective donors. **Fig. S5.** The relative abundance of cytomegalovirus reads in patient DW, JM and donors. **Fig. S6.** The temporal dynamics of communities visualized with PCoA based on gene expression profiles. The trajectory lines connect samples across the time points of the different FMT treatments. The start of each FMT is indicated in italics and colored using “sample FMT” color code. In the“sample status” legend, the shape depicts different samples and color indicates the status. The donor sample used by each FMT is marked and colored in green. ABT, antibiotic treatment. The top 200,000 expressed genes which accounted for 92% of total mapped reads were taken into account. **Fig. S7.** The top KEGG pathways significantly differentially regulated between health and pouchitis in our current study (A) and between health and ulcerative colitis (non-UC and UC) from the human microbiome project (B). The size of the dots depicts the number of differentially expressed KO genes. The color shows the adjusted p-value (FDR). The x-axis gene ratio represents the number of differentially expressed KO genes in the given pathway divided by the total number of differentially expressed KO genes. The pathways in red are shared by panel A and B. **Fig. S8.** The KEGG modules significantly altered in pouchitis (A) and UC (B) in comparison to health. The modules in red were enriched in both datasets. **Fig. S9.** Butyrate gene expression in donors and patients before FMT treatment. The relative expression levels of the genes were scaled with z-score by row. **Fig. S10.** Expression of DE butyrate synthesis genes between health and pouchitis in all samples. Butyrate synthesis genes that were differentially expressed in pouchitis (Table S1 sheet 10) were extracted from the total transcriptome and their expression levels during FMT are shown here. The barplot on the top shows the mean of relative expression of all differentially expressed butyrate genes in that sample (normalized by the total number of mapped reads per sample). Color codes depict the contributing genera, pathways and genes. The values in the heatmap show the z-score scaled relative expression of all genes of each genus. **Fig. S11.** Expression of DE genes involved in bile acids metabolism between health and pouchitis in all samples. **Fig. S12.** Expression of DE vitamin B12 synthesis genes between health and pouchitis in all samples. **Fig. S13.** Expression of DE vitamin B6 synthesis genes between health and pouchitis in all samples. **Fig. S14.** Expression of DE genes involved in tryptophan metabolism between health and pouchitis in all samples. **Fig. S15.** The expression of all DE genes grouped by genus. The barplot on the top shows the sum of the expression of all genera’s genes, while the right side barplot depicts the number of DE genes for each genus. All genera with relative read abundance >=0.1% are shown. **Fig. S16.** Expression of all genes involved in tryptophan metabolism grouped by genus. **Fig. S17.** Relative expression levels of COG categories in *F. prausnitzii* during FMT. The relative expression was calculated with total read count of a given COG category divided by the total read count of *F. prausnitzii*.

**Additional file 2: Table S1. **All sheets are listed below. Sheet 1. Clinical metadata. Sheet 2. Sample description. Sheet 3. The reads mapping statistics. Sheet 4. The read count on phylum. Sheet 5. The read count on genus. Sheet 6. The expression of Cytomegalovirus genes. Sheet 7. Differentialy expressed genes between donor and pouchitis. Sheet 8. KEGG pathways and modules enrichment in donor compared to pouchitis. Sheet 9. KEGG pathways and modules enriched in health compared to UC in HMP2 IBDM dataset. Sheet 10. DE butyrate biosysthesis genes between donor and pouchitis. Sheet 11. DE bile acid metabolism genes. Sheet 12. DE vitamin B12 biosysthesis genes. Sheet 13. DE vitamin B6 biosysthesis genes. Sheet 14. DE tryptophan metabolism genes. Sheet 15. eggNOG gene expression changes in *F. prausnitzii.*

## Data Availability

All the sequencing data is available in European Nucleotide Archive (ENA) via accession number PRJEB48996.

## Abbreviations

FMT: Fecal microbiota transplantation
CDI: *Clostridioides difficile* Infection
IBD: Inflammatory bowel disease
UC: Ulcerative colitis
IPAA: Ileal pouch-annal anastomosis
ABT: Antibiotic treatment
PCoA: Principal coordinates analysis
DE: Differential expression/expressed
CLR: Centered log ratio
IGC: Integrated gene catalog
HCMV: Human cytomegalovirus
