# Supplementary material for: Engraftment of essential functions through multiple fecal microbiota transplants in chronic antibiotic-resistant pouchitis—a case study using metatranscriptomics
Source: Microbiome. 2023 Dec 1;11:269. doi: 10.1186/s40168-023-01713-9 (PMC10691019; doi:10.1186/s40168-023-01713-9)
Supplement: Supplementary file 2 — Additional file 1: Fig. S1. Reference database construction and metatranscriptome data analysis workflows. (A) The construction of the gene database based on IGC for function and taxonomy assignment. Kraken BVAHPF represents the Kraken database built from NCBI genomes of bacteria, viruses, archaea, human, plasmids and fungi. (B) The metatranscriptome data analysis workflow. Fig. S2. Taxonomic composition of the expressed genes (transcripts) on genus (left) and species (right) level for patient CG (A), DW (B) and JM (C). The top 12 most active genera for each patient are shown. Those genera contributed over 90% of taxonomically assigned reads. Relative abundance was normalized by the total number of mapped reads for each sample. The portion of the bar which was left blank represents genera with an average abundance <1% and unknown taxa. For the species level, only the top 17 species with highest average transcript abundance are shown. Fig. S3. Composition of communities of patients CG, DW and JM based on 16S rRNA gene sequencing (genus level). Fig. S4. Cumulative rank abundance curve on the level of genus for patient CG (A), DW (B), JM (C) and their respective donors. Fig. S5. The relative abundance of cytomegalovirus reads in patient DW, JM and donors. Fig. S6. The temporal dynamics of communities visualized with PCoA based on gene expression profiles. The trajectory lines connect samples across the time points of the different FMT treatments. The start of each FMT is indicated in italics and colored using “sample FMT” color code. In the“sample status” legend, the shape depicts different samples and color indicates the status. The donor sample used by each FMT is marked and colored in green. ABT, antibiotic treatment. The top 200,000 expressed genes which accounted for 92% of total mapped reads were taken into account. Fig. S7. The top KEGG pathways significantly differentially regulated between health and pouchitis in our current study (A) and between health and ul [file 40168_2023_1713_MOESM1_ESM.docx]

**Supplementary Information**

**Table S1. All sheets are listed below.**

Sheet 1. Clinical metadata

Sheet 2. Sample description

Sheet 3. The reads mapping statistics

Sheet 4. The read count on phylum

Sheet 5. The read count on genus

Sheet 6. The expression of Cytomegalovirus genes

Sheet 7. Differentialy expressed genes between donor and pouchitis

Sheet 8. KEGG pathways and modules enrichment in donor compared to pouchitis

Sheet 9. KEGG pathways and modules enriched in health compared to UC in HMP2 IBDM dataset

Sheet 10. DE butyrate biosysthesis genes between donor and pouchitis

Sheet 11. DE bile acid metabolism genes

Sheet 12. DE vitamin B12 biosysthesis genes

Sheet 13. DE vitamin B6 biosysthesis genes

Sheet 14. DE tryptophan metabolism genes

Sheet 15. eggNOG gene expression changes in *F. prausnitzii*

**
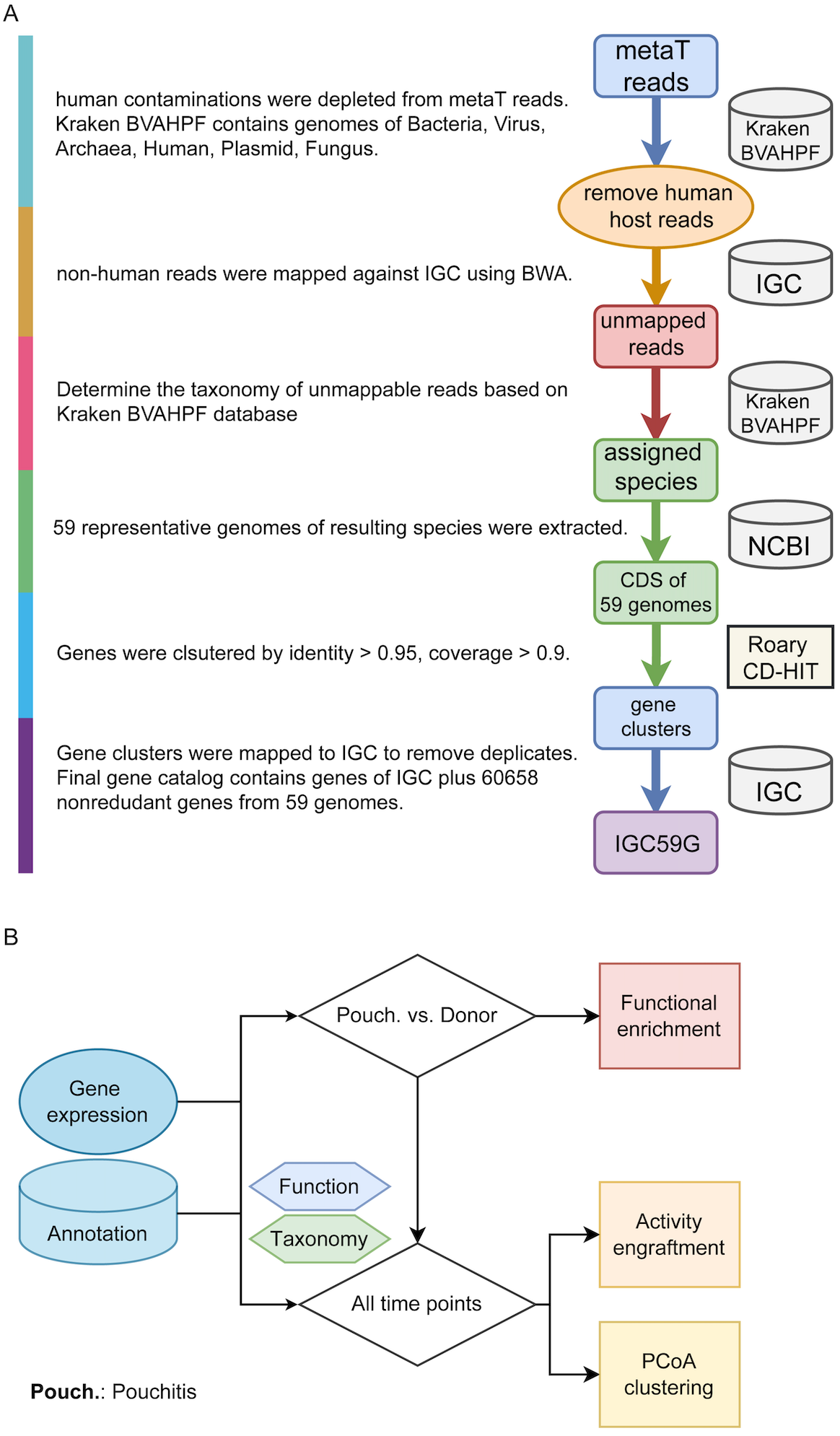
**

**Figure S1. Reference database construction and metatranscriptome data analysis workflows.** (**A**) The construction of the gene database based on IGC for function and taxonomy assignment. Kraken BVAHPF represents the Kraken database built from NCBI genomes of bacteria, viruses, archaea, human, plasmids and fungi. (**B**) The metatranscriptome data analysis workflow.


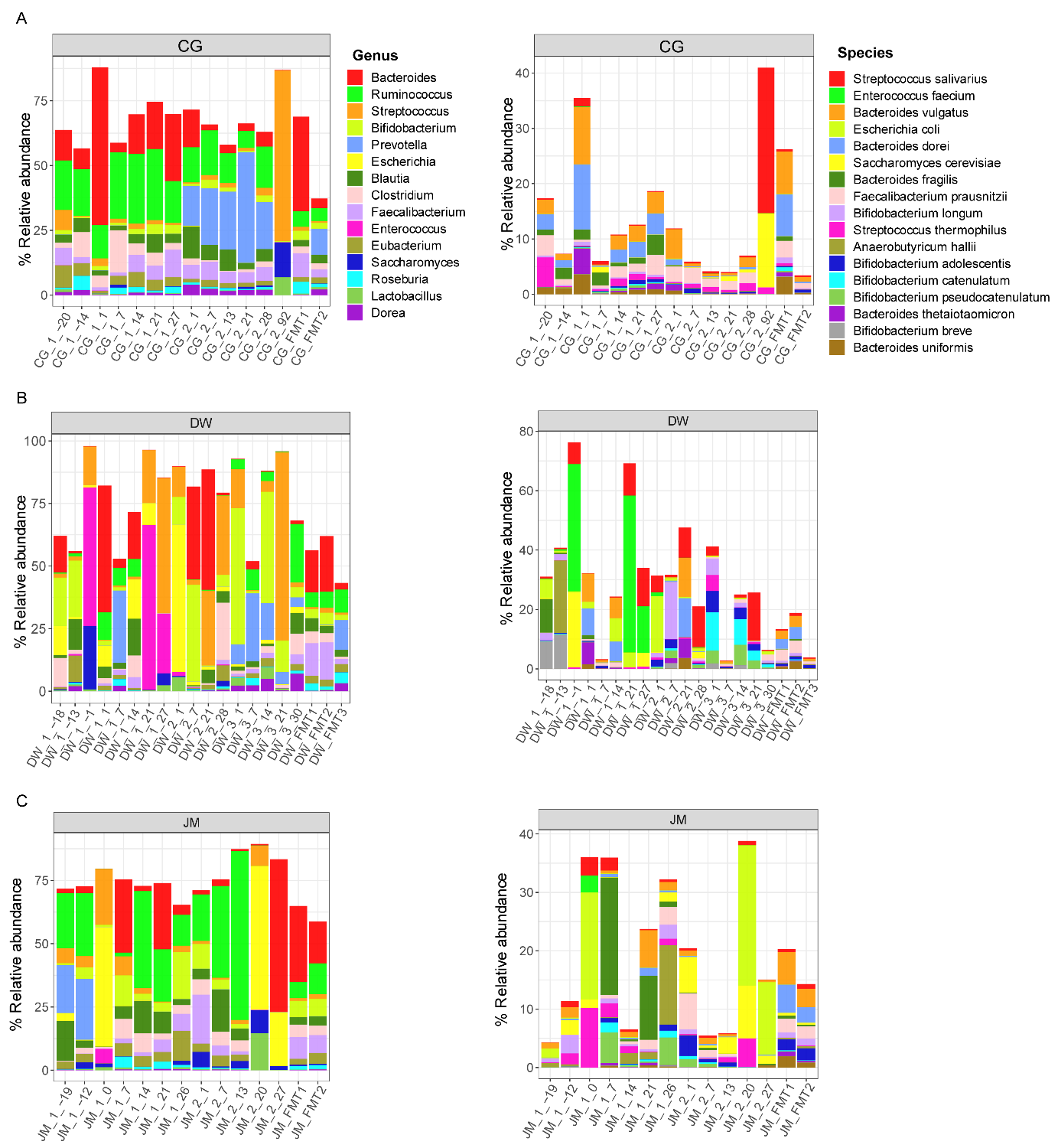


**Figure S2.** **Taxonomic composition of the expressed genes (transcripts) on genus (left) and species (right) level for patient CG (A), DW (B) and JM (C).** The top 12 most active genera for each patient are shown. Those genera contributed over 90% of taxonomically assigned reads. Relative abundance was normalized by the total number of mapped reads for each sample. The portion of the bar which was left blank represents genera with an average abundance <1% and unknown taxa. For the species level, only the top 17 species with highest average transcript abundance are shown.


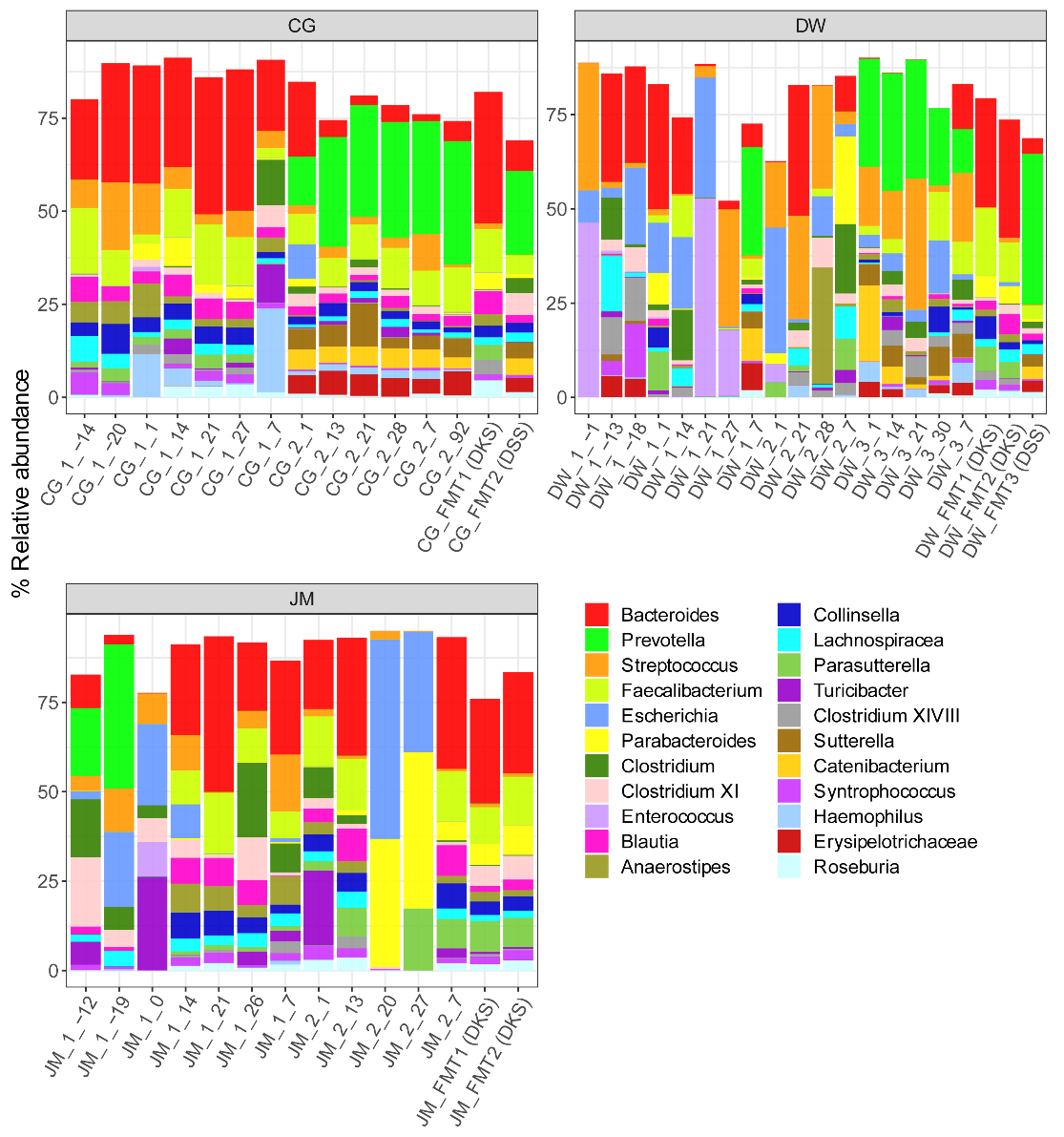


**Figure S3. Composition of communities of patients CG, DW and JM based on 16S rRNA gene sequencing (genus level).**

**
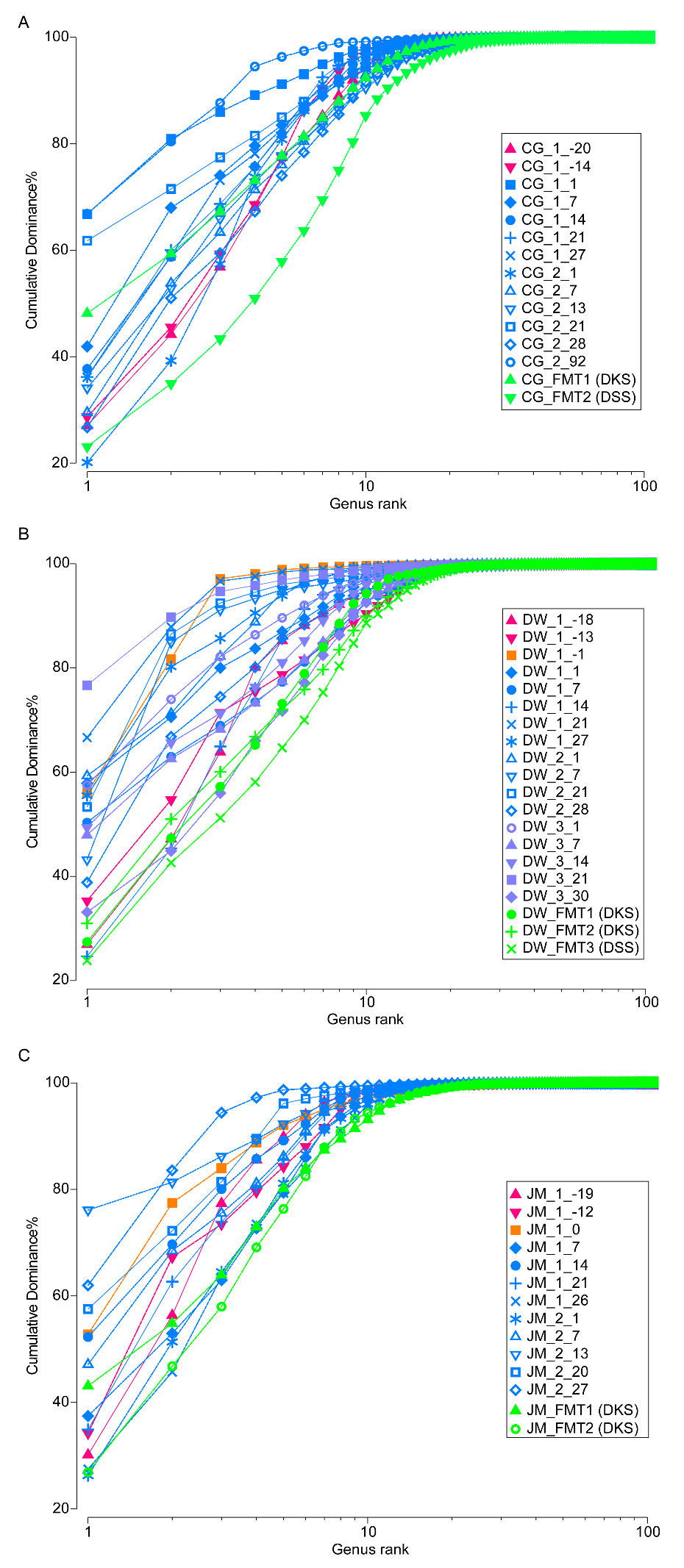
**

**Figure S4. Cumulative rank abundance curve on the level of genus for patient CG (A), DW (B), JM (C) and their respective donors.**


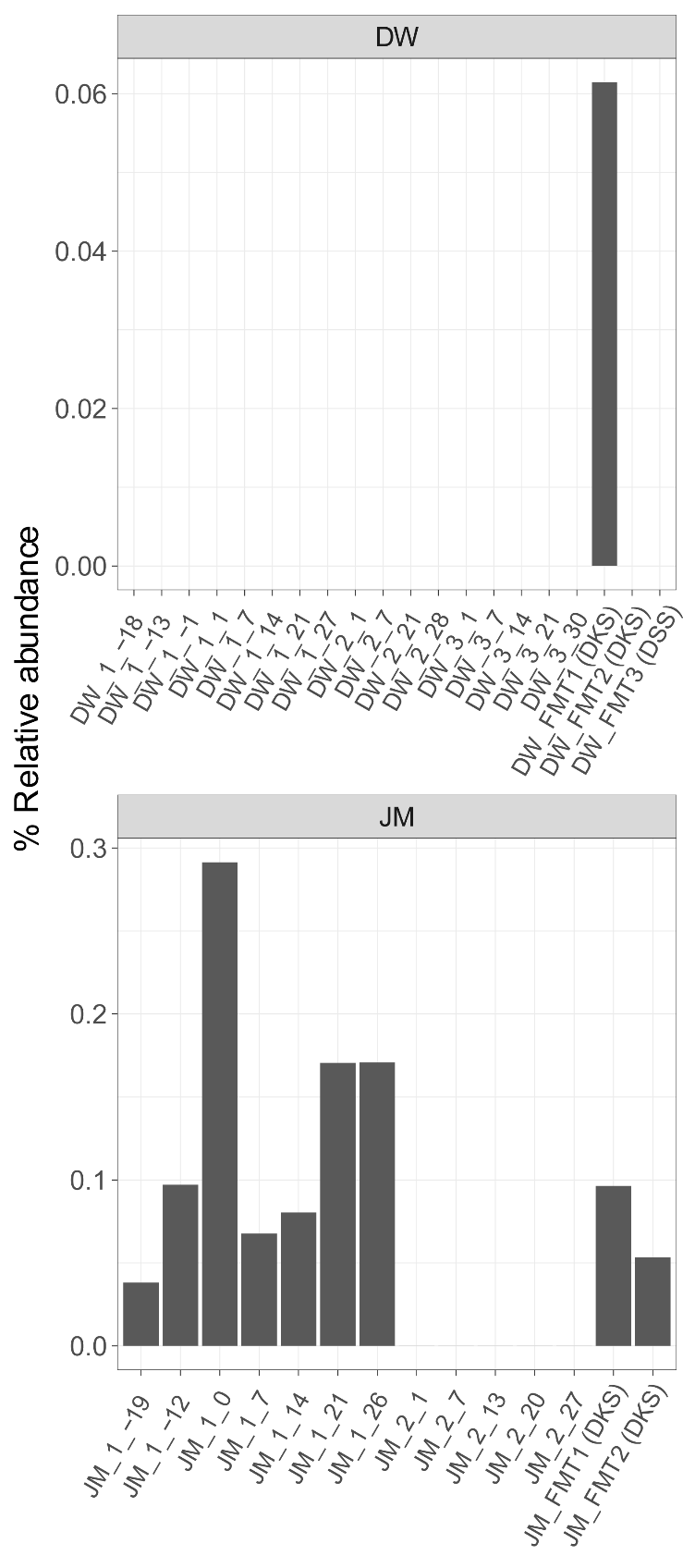


**Figure S5. The relative abundance of cytomegalovirus reads in patient DW, JM and donors.**

**
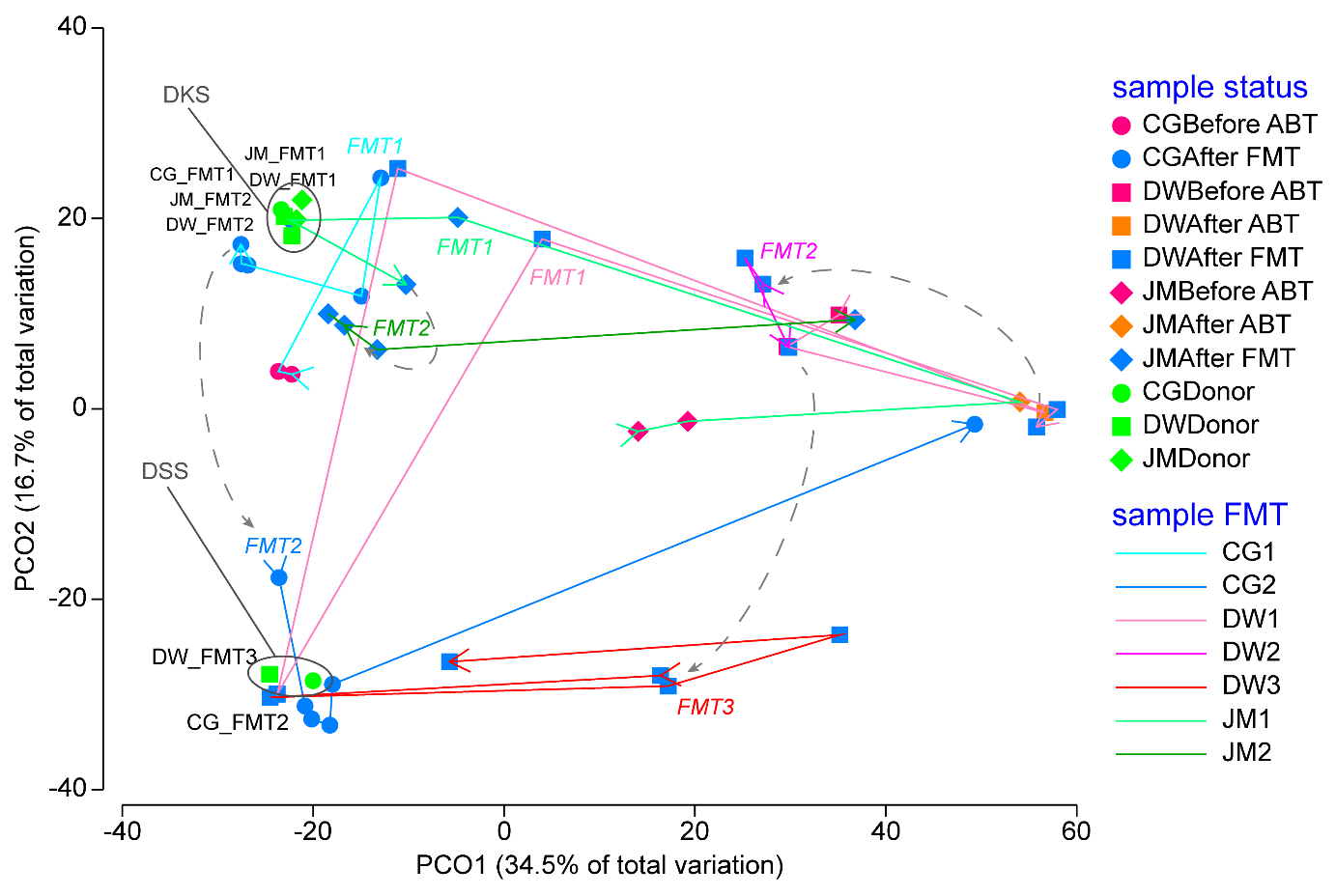
**

**Figure S6. The temporal dynamics of communities visualized with PCoA based on gene expression profiles.**  The trajectory lines connect samples across the time points of the different FMT treatments. The start of each FMT is indicated in italics and colored using “sample FMT” color code. In the “sample status” legend, the shape depicts different samples and color indicates the status. The donor sample used by each FMT is marked and colored in green. ABT, antibiotic treatment. The top 200,000 expressed genes which accounted for 92% of total mapped reads were taken into account.


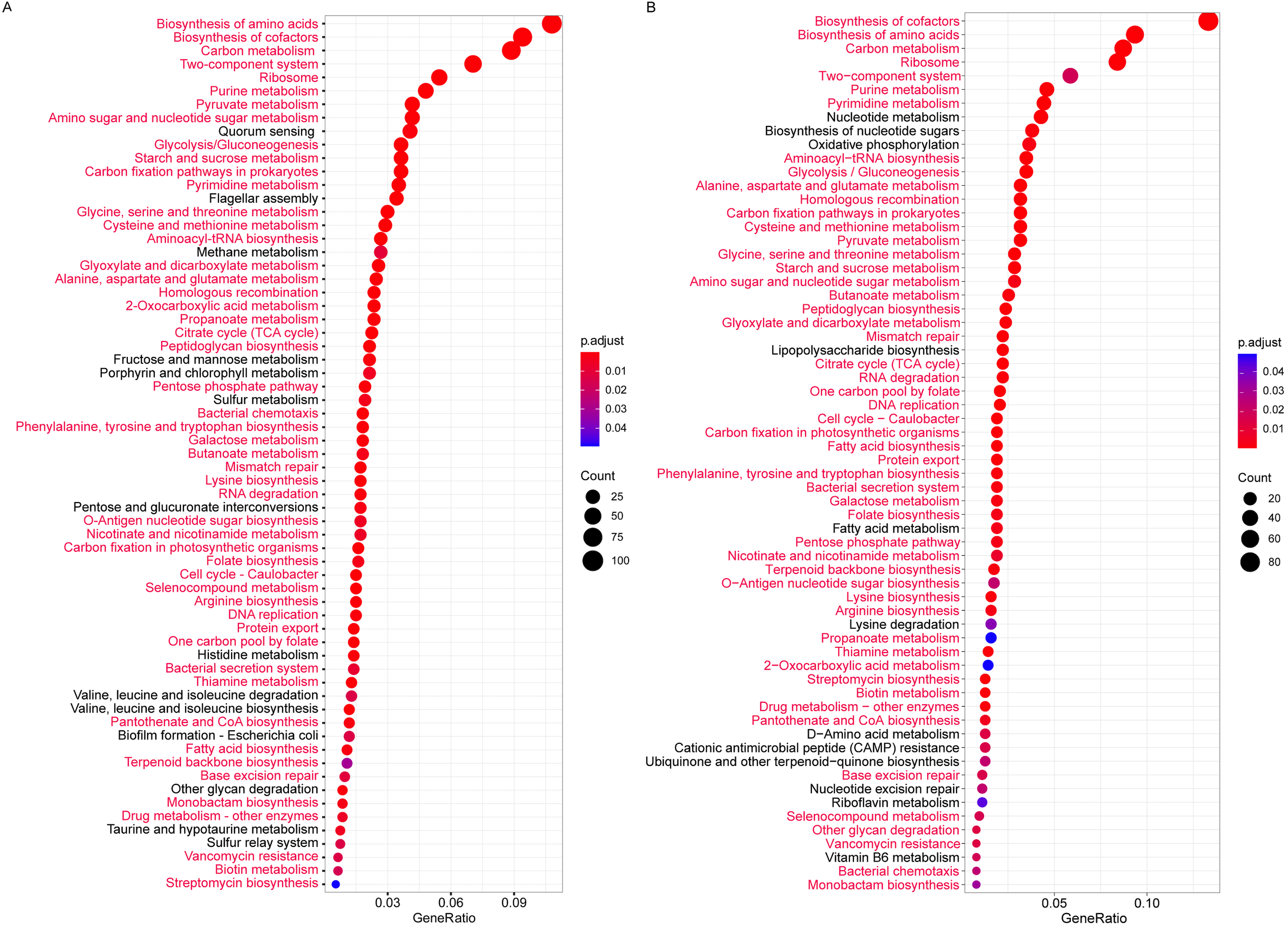


**Figure S7. The top KEGG pathways significantly differentially regulated between health and pouchitis in our current study (A) and between health and ulcerative colitis (non-UC and UC) from the human microbiome project (B).** The size of the dots depicts the number of differentially expressed KO genes. The color shows the adjusted p-value (FDR). The x-axis gene ratio represents the number of differentially expressed KO genes in the given pathway divided by the total number of differentially expressed KO genes. The pathways in red are shared by panel A and B.


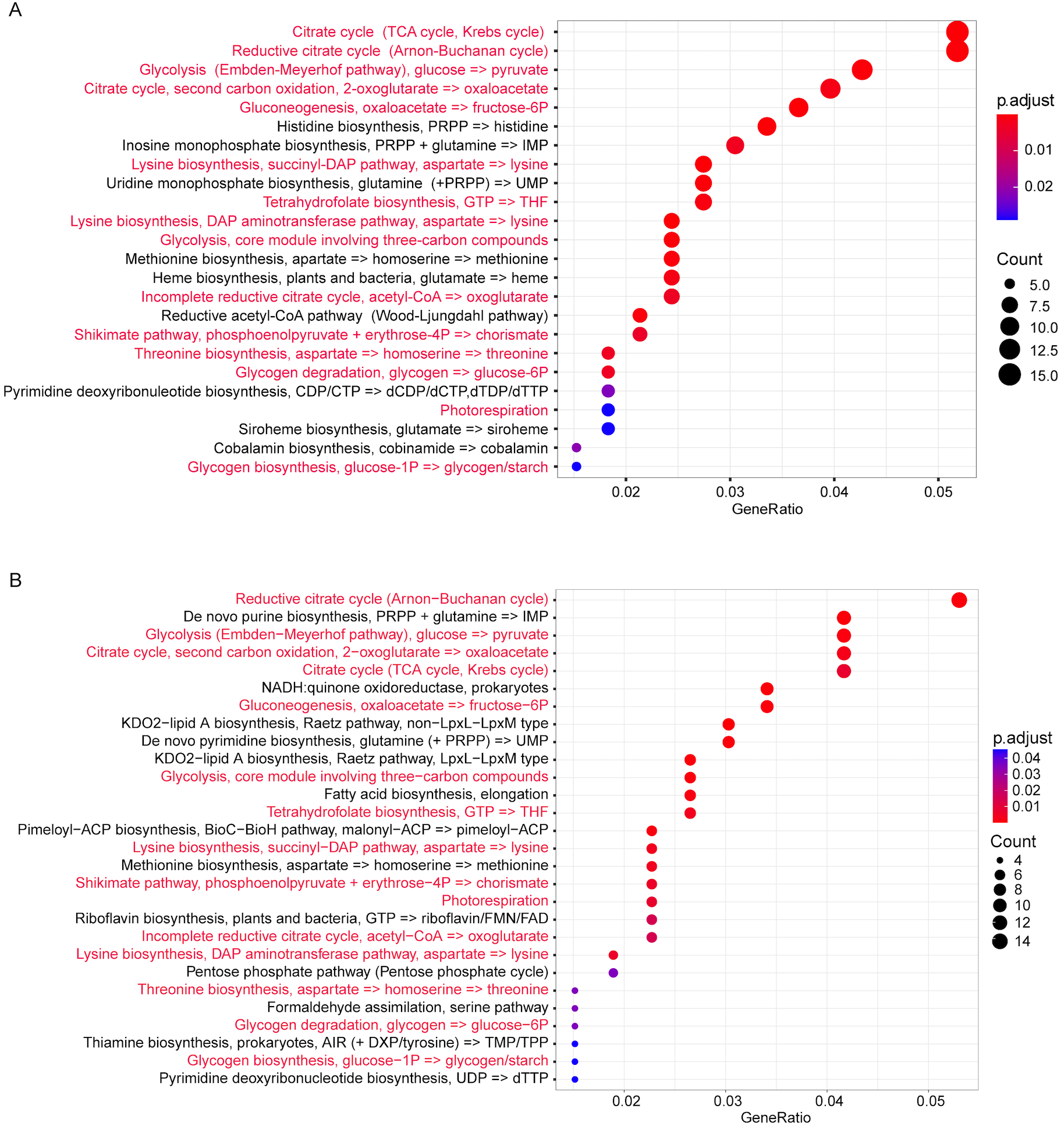


**Figure S8. The KEGG modules significantly altered in pouchitis (A) and UC (B) in comparison to health.** The modules in red were enriched in both datasets.

**
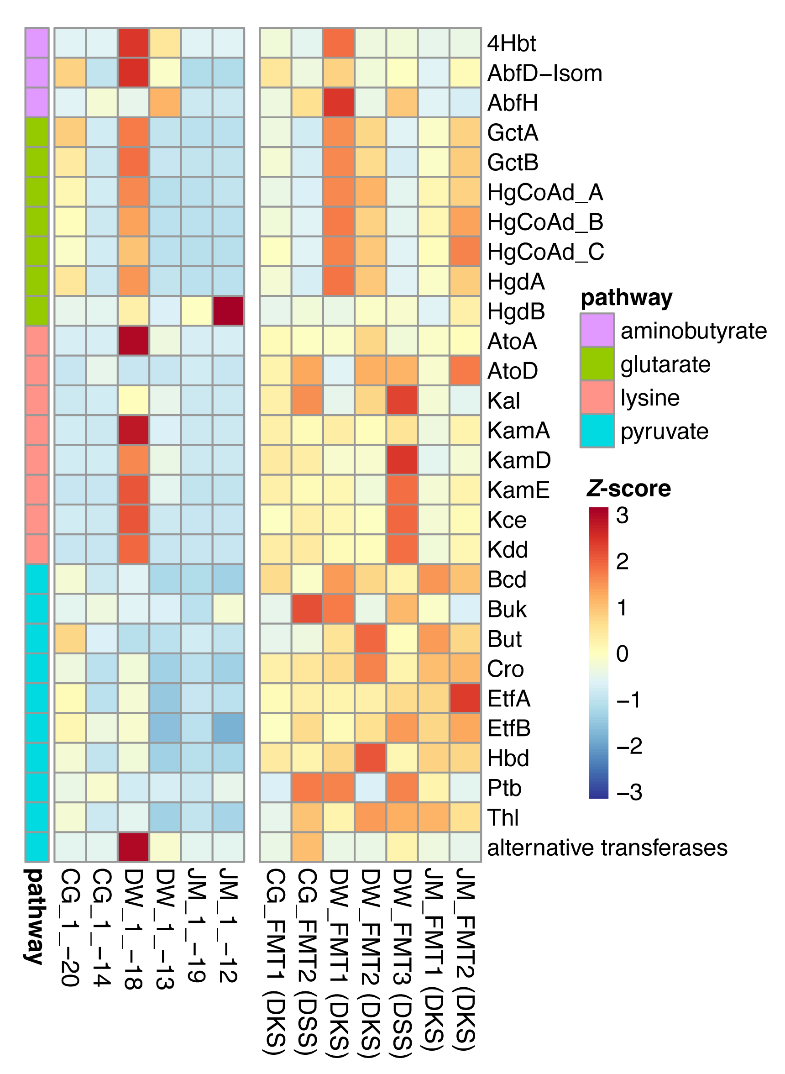
**

**Figure S9. Butyrate gene expression in donors and patients before FMT treatment.** The relative expression levels of the genes were scaled with z-score by row.

**
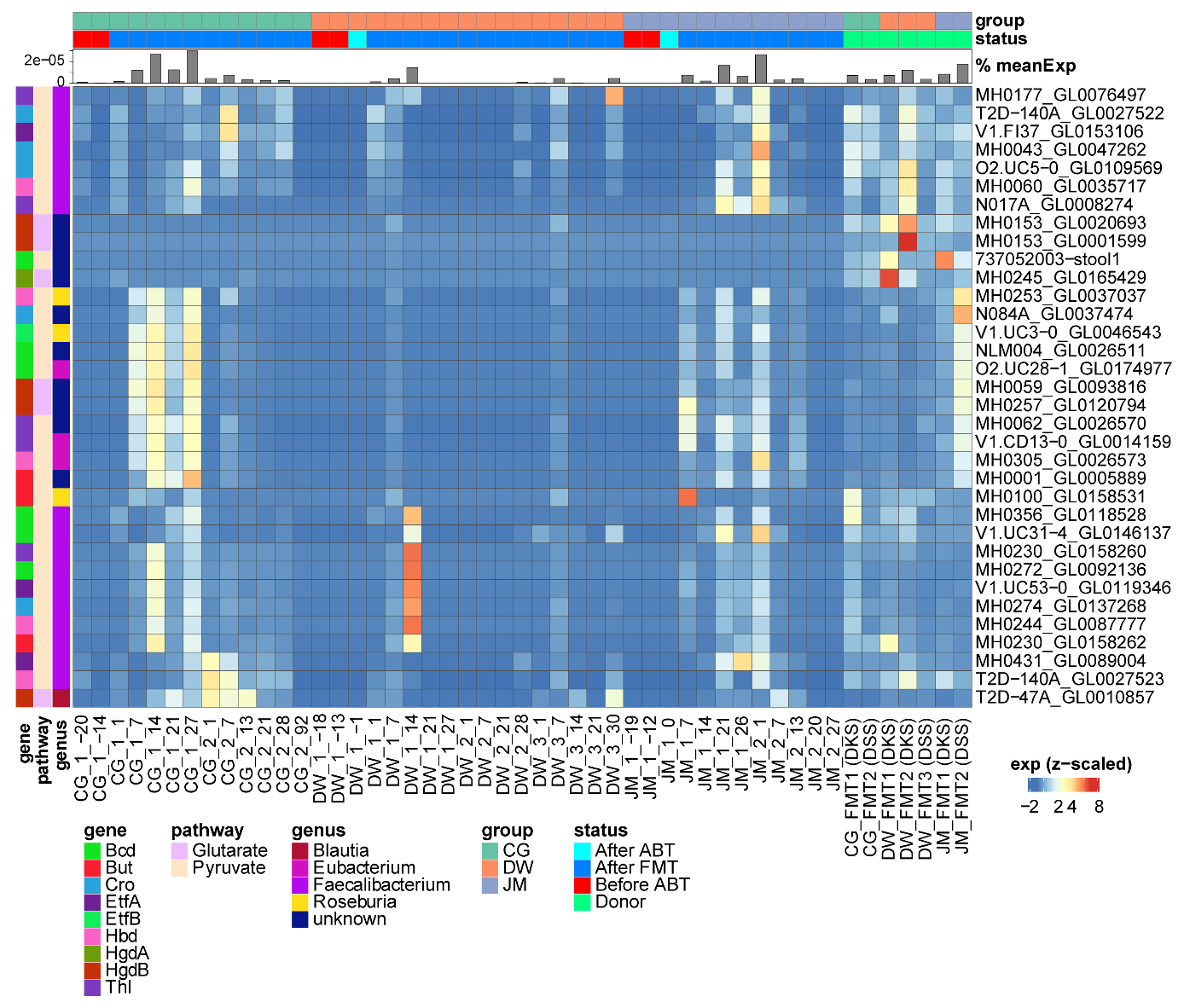
**

**Figure S10. Expression of DE butyrate synthesis genes between health and pouchitis in all samples.** Butyrate synthesis genes that were differentially expressed in pouchitis (Table S1 sheet 10) were extracted from the total transcriptome and their expression levels during FMT are shown here. The barplot on the top shows the mean of relative expression of all differentially expressed butyrate genes in that sample (normalized by the total number of mapped reads per sample). Color codes depict the contributing genera, pathways and genes. The values in the heatmap show the z-score scaled relative expression of all genes of each genus.

**
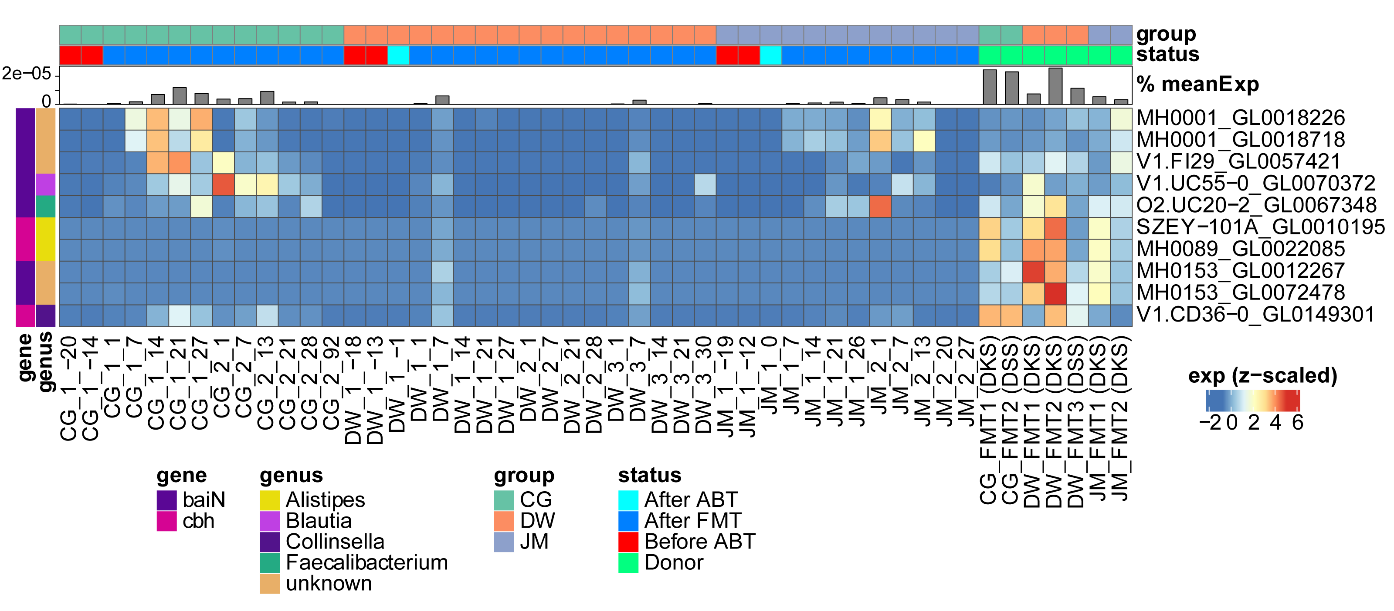
**

**Figure S11. Expression of DE genes involved in bile acids metabolism between health and pouchitis in all samples.**

**
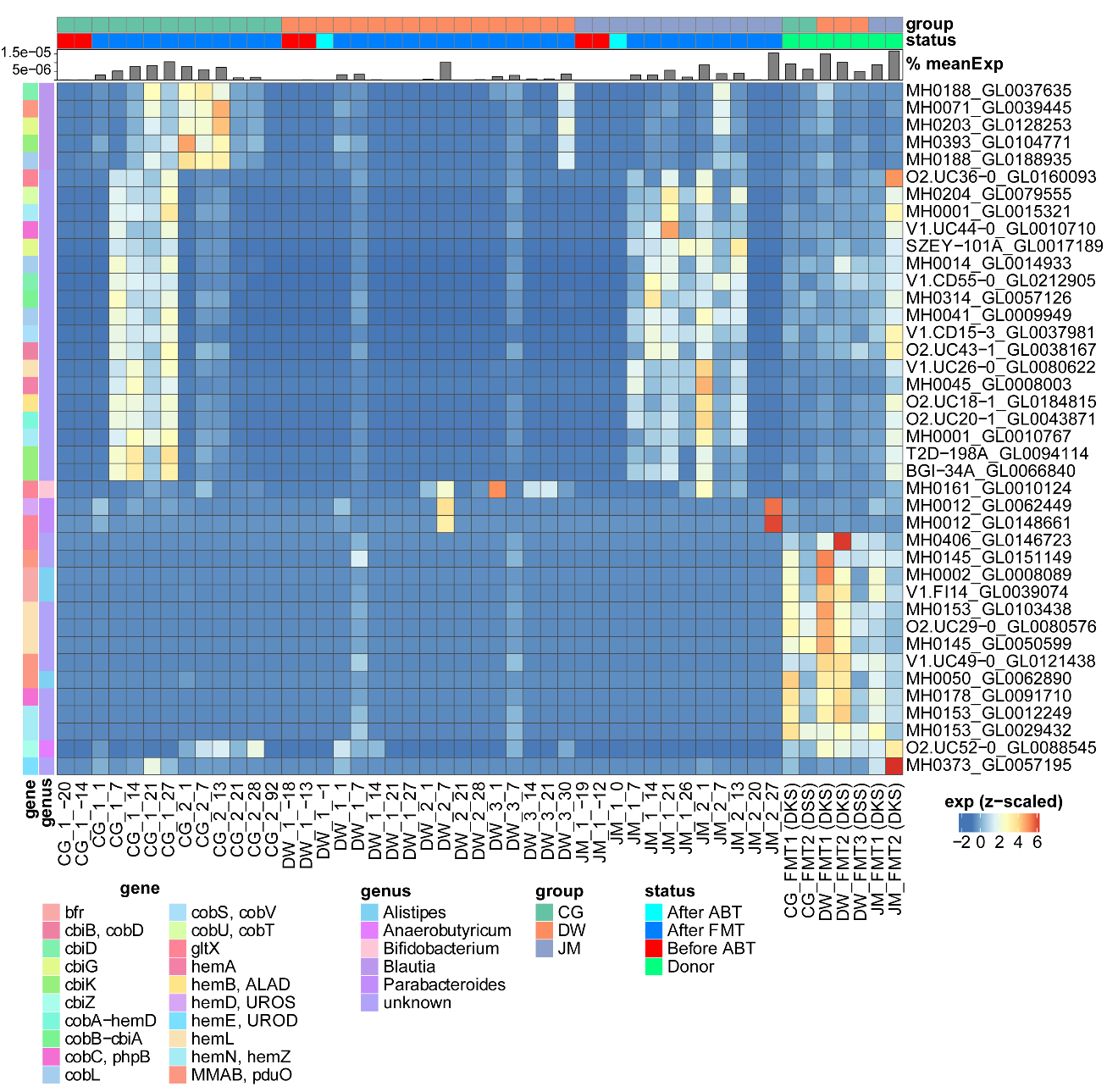
**

**Figure S12. Expression of DE vitamin B12 synthesis genes between health and pouchitis in all samples.**

**
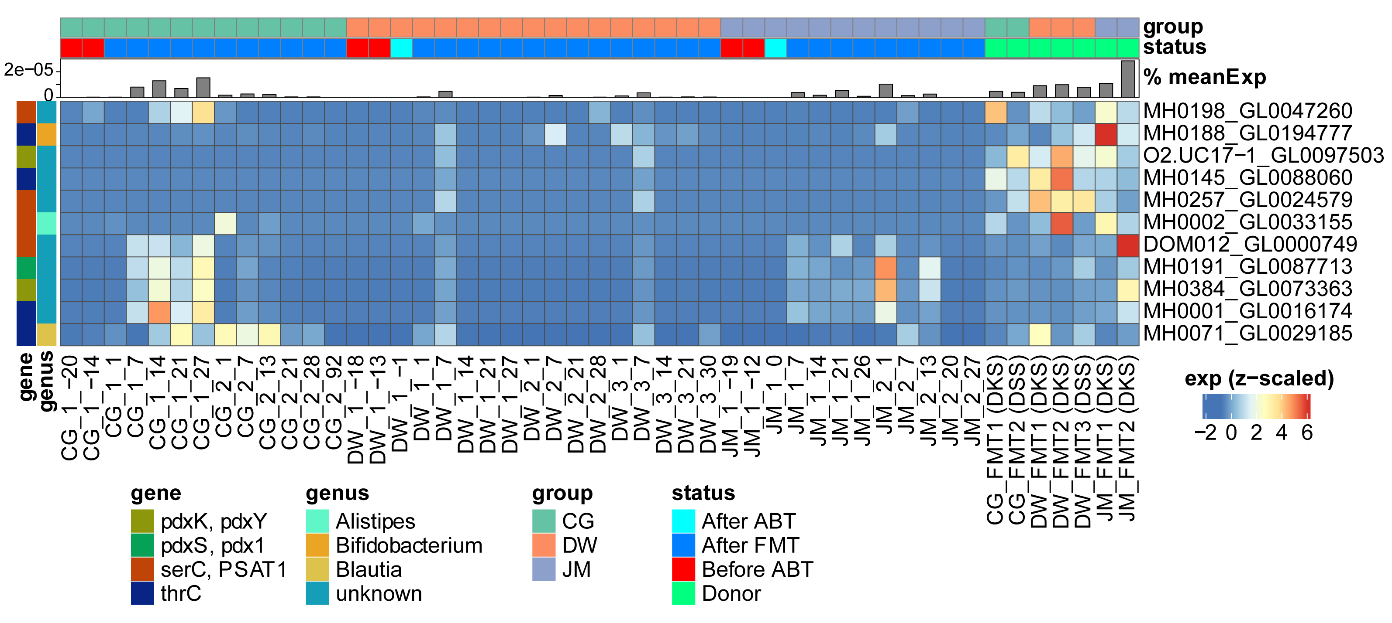
**

**Figure S13. Expression of DE vitamin B6 synthesis genes between health and pouchitis in all samples.**

**
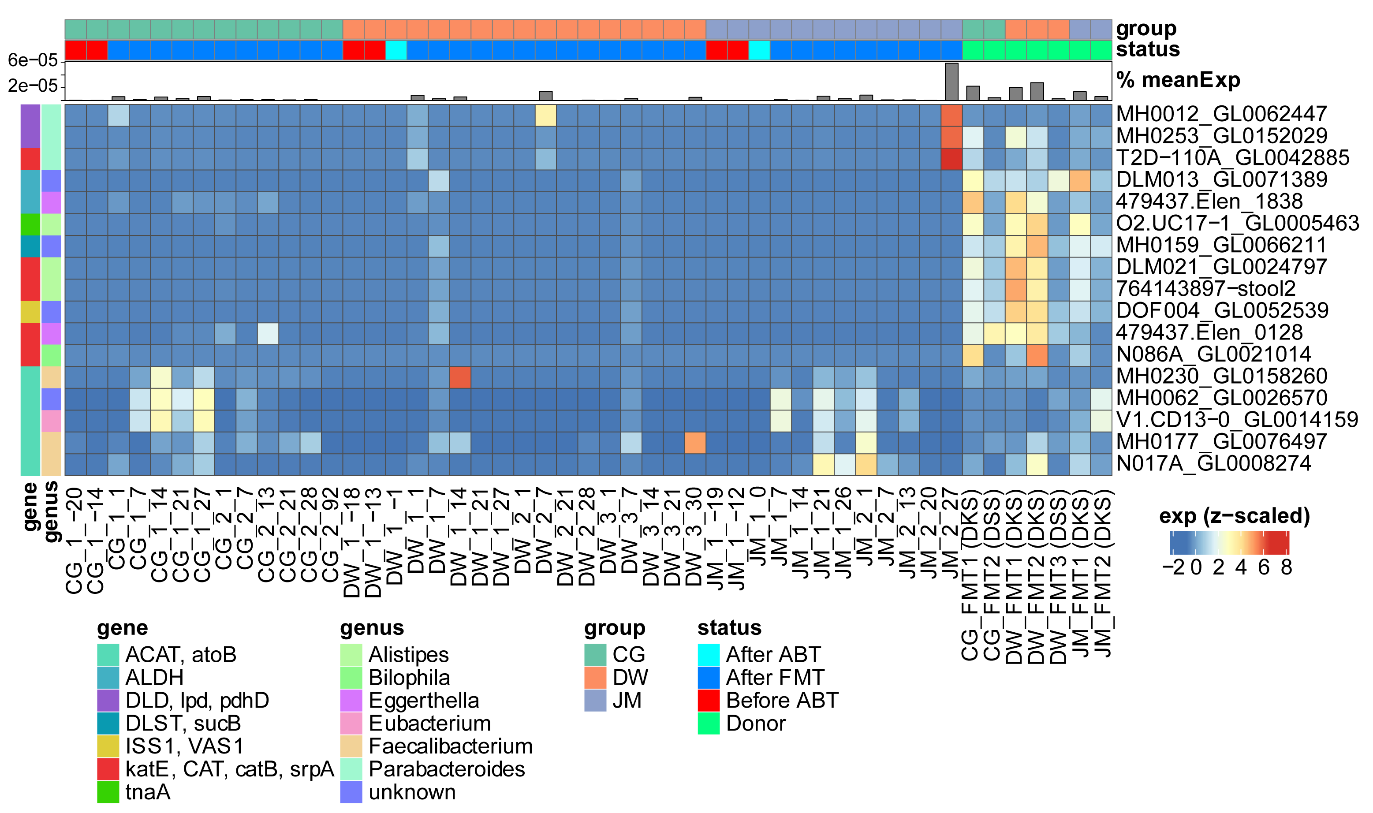
**

**Figure S14. Expression of DE genes involved in tryptophan metabolism between health and pouchitis in all samples.**

**
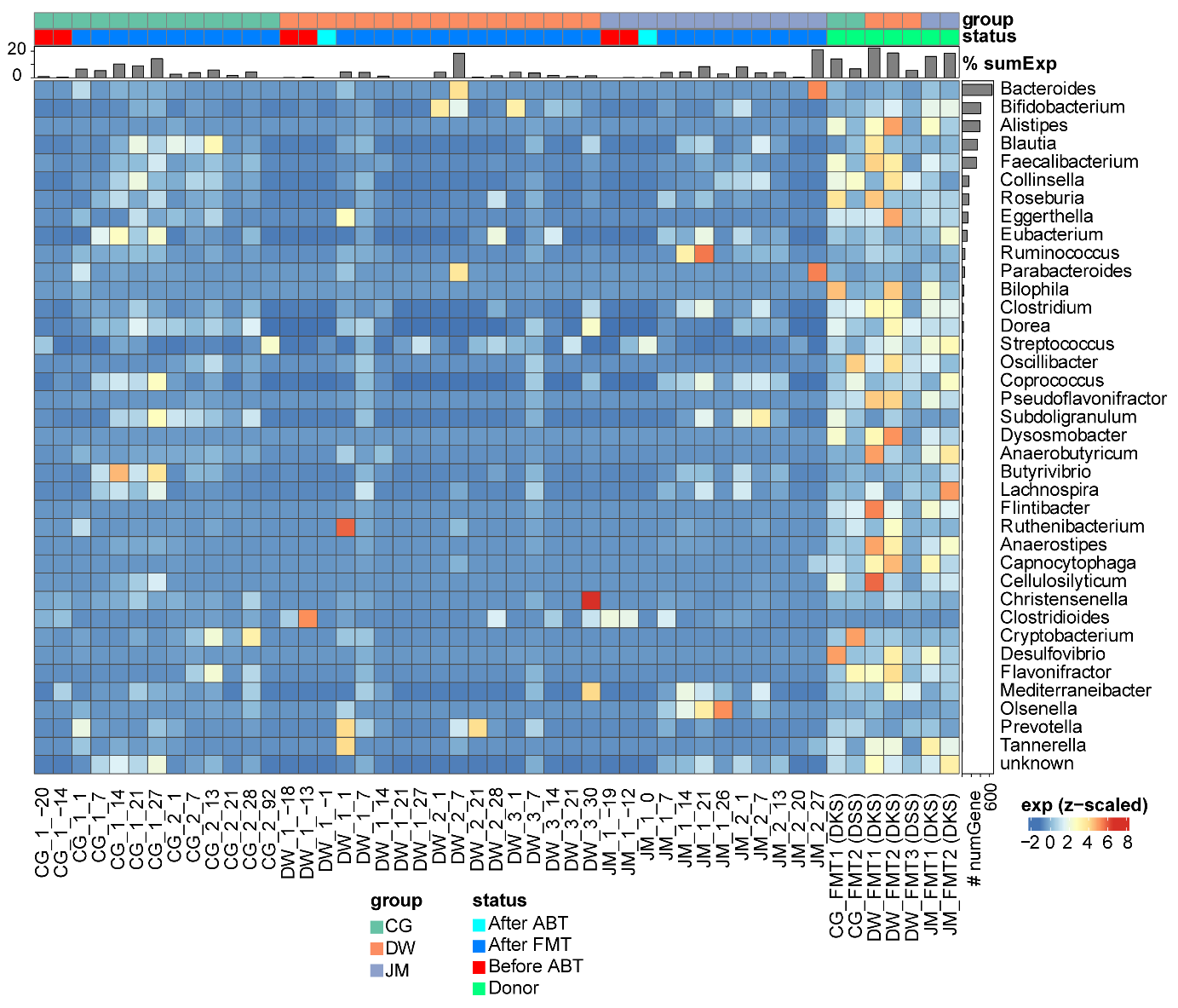
**

**Figure S15**. **The expression of all DE genes grouped by genus.** The barplot on the top shows the sum of the expression of all genera’s genes, while the right side barplot depicts the number of DE genes for each genus. All genera with relative read abundance >=0.1% are shown.

**
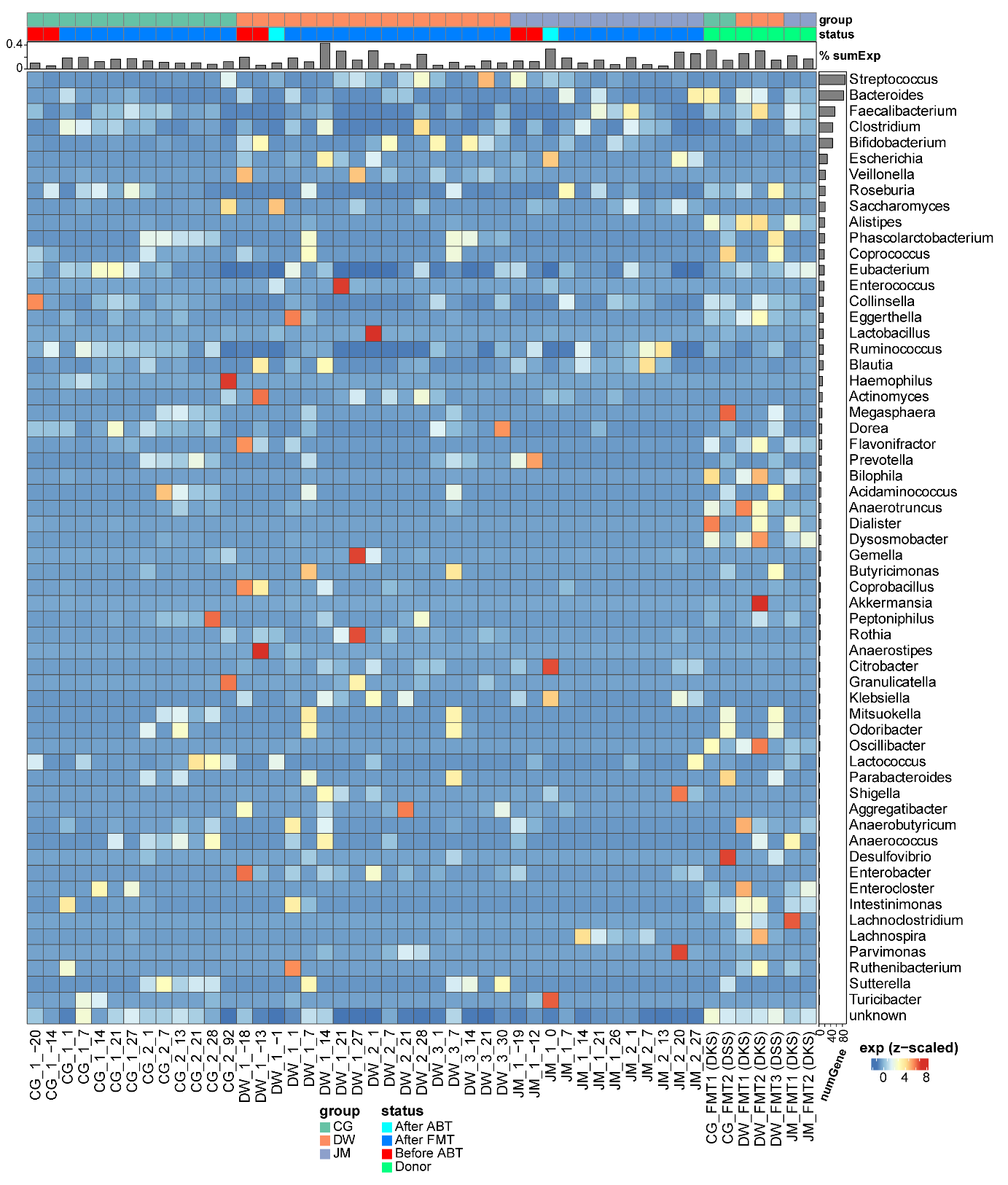
**

**Figure S16. Expression of all genes involved in tryptophan metabolism grouped by genus.**


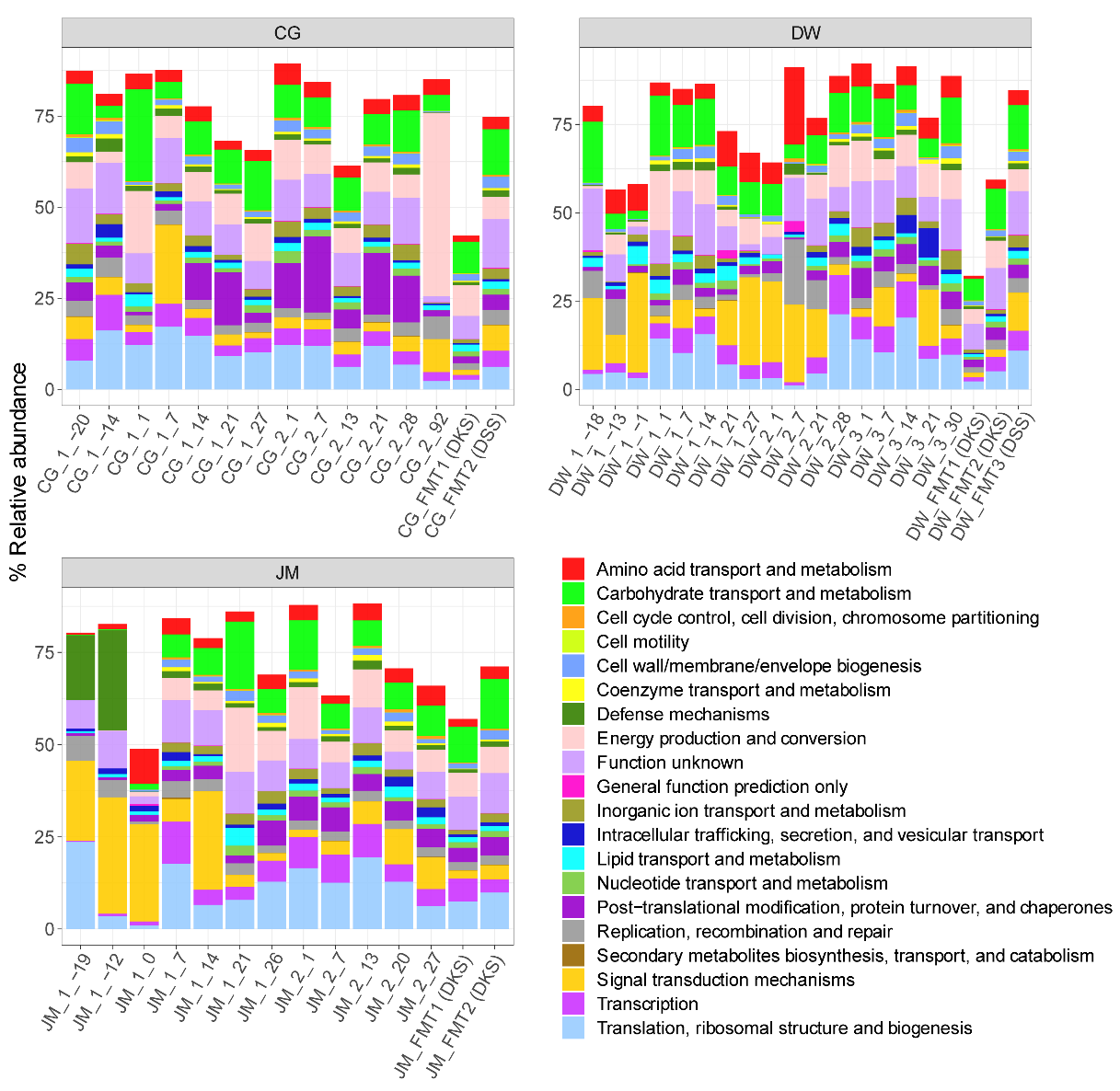


**Figure S17. Relative expression levels of COG categories in *F. prausnitzii* during FMT.** The relative expression was calculated with total read count of a given COG category divided by the total read count of *F. prausnitzii*.
